# Enhancing apoptosis-mediated anticancer activity of evodiamine through protein-based nanoparticles in breast cancer cells

**DOI:** 10.1038/s41598-024-51970-3

**Published:** 2024-01-31

**Authors:** Raghu Solanki, Pradeep Kumar Rajput, Bhavana Jodha, Umesh C. S. Yadav, Sunita Patel

**Affiliations:** 1 School of Life Sciences, Central University of Gujarat, Gandhinagar, 382030 India; 2 Special Centre for Medicine and Special Centre for Systems Medicine, Jawaharlal Nehru University, New Delhi, 110067 India

**Keywords:** Biochemistry, Biological techniques, Cancer, Molecular biology, Biomarkers, Diseases, Medical research, Materials science, Nanoscience and technology

## Abstract

In the cutting-edge era of developing precision therapeutics, nanoparticles have emerged as a potent drug delivery system. Altering the size of poorly water-soluble drugs to nanoscale could confer change in their physical properties, including enhanced water solubility and bioavailability. Evodiamine (EVO), a natural indolequinone alkaloid extract from *Evodia rutaecarpa*, has shown several important pharmacological applications, anti-cancer being one of them. Protein-based nano-drug delivery systems have gained the interest of researchers due to their better biocompatibility, biodegradability, non-immunogenicity and non-toxicity. In the present study, EVO encapsulated BSA nanoparticles (ENPs) were synthesized and characterized, which were nanoscale-sized (~ 150 nm), monodispersed, spherical shaped, and showed high entrapment efficiency (~ 86%) and controlled drug release. The *in-vitro* anti-cancer activity of ENPs on human breast cancer cells was dose- and time-dependent. The apoptotic molecular mechanism investigated using FACS, qRT-PCR, and western blotting analysis, revealed increased expression of p53 and Bax and decreased expression of Bcl-2. Biological studies demonstrated comparatively more efficient and targeted delivery of ENPs than pure EVO. The comprehensive physiochemical characterization and in-vitro validation collectively pinpoint ENPs as a promising avenue for harnessing the therapeutic potential of the natural anti-cancer compound EVO. The findings indicate improved cytotoxicity, positioning ENPs as a propitious strategy for advancing breast cancer treatment.

## Introduction

Breast cancer is the most common malignancy in women with a high incidence and mortality rate. According to GLOBOCAN 2020, there were over 2.3 million new cases with 24.5% and 0.6 million deaths, of which 15.5% happened among all cancer types in women^1^. Because of population growth and aging alone, the burden of breast cancer is expected to rise to almost 3 million new cases and 1 million deaths annually by 2040^2^. Chemically synthesized drugs have been used extensively for breast cancer therapy. However, non-selectivity between normal cells and cancer cells and multidrug resistance are still challenges for chemotherapeutic agents^3^. Thus, new strategies and novel chemoprevention agents are urgently needed. Phytochemicals derived from medicinal plants, serve as novel drugs for breast cancer treatment^4^ and using novel delivery tools for enhanced bioavailability and targeted drug delivery is the need of the hour.

Evodiamine (EVO) is an indolequinone alkaloid derived from *Evodia rutaecarpa*^5^*.* EVO is composed of a pentacyclic structure including three nitrogen atoms and exhibits abundant pharmacological potential including anti-cancer^6^, anti-bacterial^7^, and anti-neurodegenerative^8^, anti-inflammatory^9^, anti-obesity^10^, anti-depressant^11^ and cardioprotective^12^. Furthermore, reported studies demonstrated that it has a promising anticancer property by specifically inducing apoptosis and cell cycle arrest, reducing cell proliferation and inhibiting metastasis in cancer cells^13–15^. Studies have also shown that evodiamine activates pro-apoptotic signaling pathways, leading to the activation of caspases, which are the enzymes responsible for executing apoptosis^16,17^. Despite its potential anti-cancer activity, the pharmacological applications of EVO are restricted due to challenges like poor aqueous solubility, limited absorption in the gastrointestinal tract, and low oral bioavailability. Further, EVO has been reported to cause side effects such as anorexia, diarrhea, constipation, stomach discomfort and even serious problems like liver toxicity and cardiotoxicity when taken in high doses or for a prolonged period^18^. To address these challenges newer approaches such as nano-delivery vehicles are being considered for otherwise potent drug candidates.

Recently, protein-based nano-systems, especially albumin nanoparticles, have gained much attention because albumins are naturally-derived and possess several desirable properties like biodegradability, biocompatibility, non-immunogenicity, high drug binding capacity, bio-stability, low toxicity, and long circulation time^19,20^. Studies have shown that albumin nanoparticles can effectively encapsulate and deliver a variety of therapeutic agents, including small molecules, proteins, and nucleic acids, to the target sites in the body^21^. They have also been used as scaffolds for tissue engineering applications and contrast agents for imaging. Bovine serum albumins (BSA) and human serum albumins (HSA) are common albumins used in the pharmaceutical and biomedical sectors^22^. In addition, BSA is widely used as a nano-carrier to deliver several bioactive compounds and has numerous applications in biomedicine, especially because it is abundantly available, inexpensive, and simple to purify^23,24^.

In this study, EVO loaded BSA nanoparticles (ENPs) were prepared using the desolvation method. Encapsulation of EVO in BSA NPs was confirmed using different physicochemical techniques and biological studies were done *in-vitro* on human breast cancer cells (MDA-MB-231 and MCF-7 cells). Furthermore, ENPs showed greater potential than EVO and improved anticancer activity by targeting the apoptosis molecular pathway. Thus, prepared ENPs could be an ideal nanocarrier for EVO to enhance its anticancer therapeutic potential.

## Results and discussion

### Preparation and characterization of BNPs and ENPs

**Figure 1:**
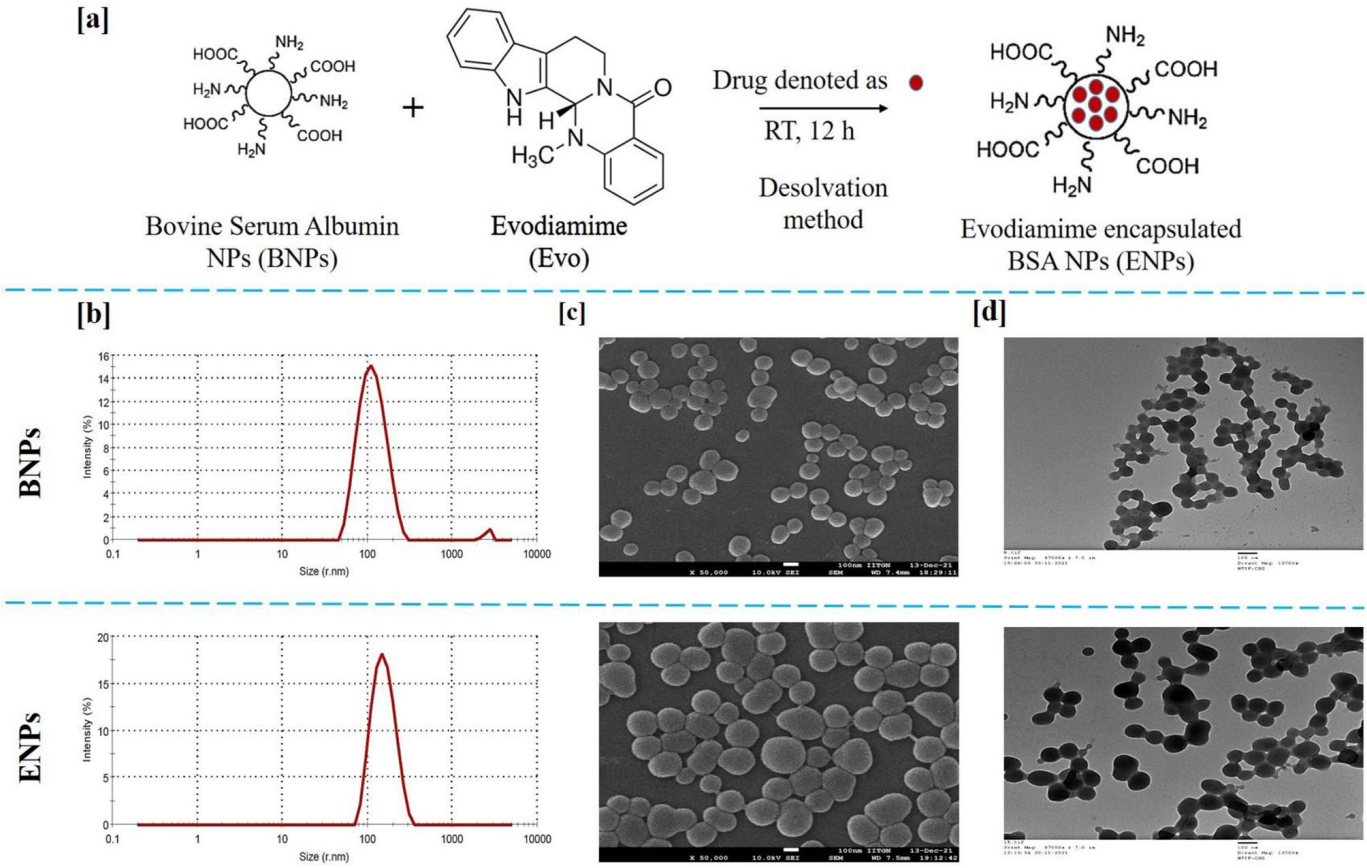
Schematic representation of the preparation of Evodiamine loaded Bovine Serum Albumin (ENPs) (**a**). The hydrodynamic size distribution was determined by DLS (**b**) FE-SEM images (**c**) and TEM images (**d**) of BNPs and ENPs.

EVO loaded nanoparticles (ENPs) and blank nanoparticles (BNPs) were successfully prepared using the desolvation method (Fig. 1a) and characterized by various physicochemical techniques. Size and shape are considered the key characteristics of any synthesized nanoparticle and these properties make them good nanocarriers as they play important roles during drug delivery in cancer cells. As per available literature, the average size below 200 nm and round-shaped nanoparticles are preferable^25^. Thus, the particle size of prepared BNPs and ENPs was measured using Dynamic Light Scattering (DLS).

Figure 1b represents the average hydrodynamic size of BNPs and ENPs. The average hydrodynamic particle size, Polydispersity Index (PDI) and Zeta Potential (ZP) were found to be 116.0 nm, 0.144 and − 26.8, respectively (Fig. 1b, Figure S1 and Table 1). The average hydrodynamic particle size, PDI and ZP of ENPs were measured 150.2 nm, 0.212 and − 36.6, respectively (Table 1). Particle size, PDI and ZP values indicated nanosized, uniform and highly monodisperse nanoparticles. From the Field Emission-Scanning Electron Microscopy (FE-SEM) images (Fig. 1c) of the prepared BNPs and ENPs it is evident that the prepared nanoparticles were smooth and round. After FE-SEM analysis, the size distribution of BNPs and ENPs was determined to be 79.28 nm and 91.83 nm, respectively (Figure S2). Transmission Electron Microscope (TEM) analysis (Fig. 1d) also corroborated with the FE-SEM results. Thus, the data from the DLS, FE-SEM and TEM demonstrated that prepared nanoparticles could be ideal nanocarriers for the delivery of EVO.

**Table 1:** Particle size, PDI, ZP, %EE and %LC of ENPs.

Prepared NPs	Hydrodynamic size (nm)	PDI	ZP	%EE	%LC
BNPs	116.0	0.144	− 26.8	–	–
ENPs	150.2	0.212	− 36.6	86.54	7.86

EVO entrapment (%EE) and EVO loading capacity (%LC) were estimated using a UV–Vis spectrophotometer and it was found to be 86.54% and 7.86%, respectively (Table 1).

**Figure 2:**
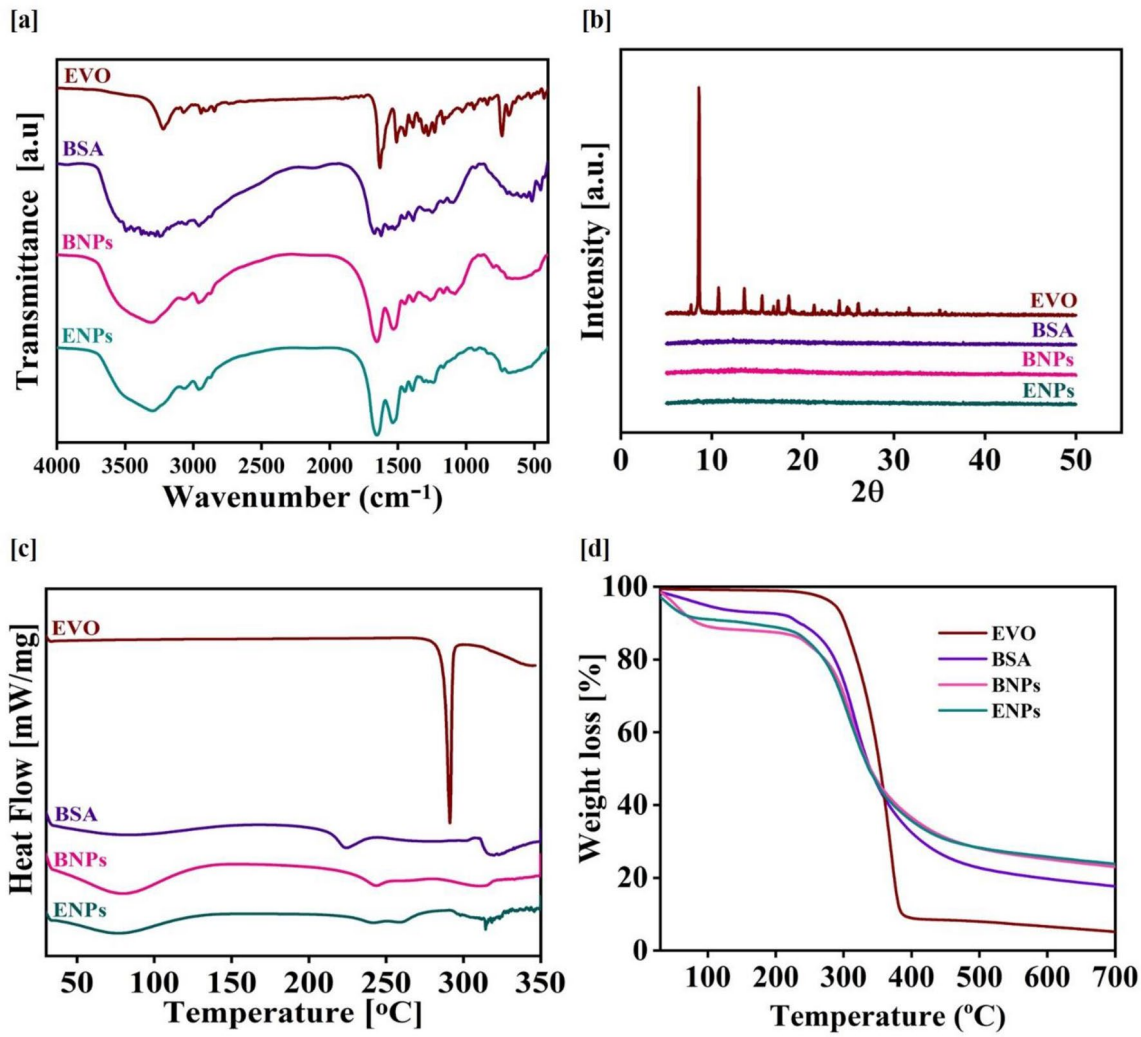
Physicochemical characterization of ENPs. FTIR spectra (**a**), XRD patterns (**b**), DSC thermograms (**c**) and TGA analysis (**d**) of pure EVO, pure BSA, BNPs and ENPs.

The Fourier Transform Infrared Spectroscopy (FTIR) was performed to confirm the encapsulation of EVO within BNPs. As shown in Fig. 2a, FTIR analysis of pure EVO spectra revealed the absorption peak at 3224 cm^−1^, defining the peak of –NH group. A characteristic functional group of pure EVO, cyclobenzene, was also present as peaks observed at 1509, 1281 and 735 cm^−1^.

The –CH_3_ and –CH_2_ groups were related to the distinctive peaks at 2945 and 2843 cm^−1^, respectively. The carbonyl group's distinctive absorption peak was located at 1606 cm^−1^. FTIR spectra analysis of pure EVO was also corroborated by a reported study^26^. The evidence of amide I and II was confirmed by the BSA spectrum's significant peaks at 1640 and 1510 cm^−1^, which also supported earlier findings^27^. The FTIR spectra of prepared ENPs showed significant peaks at 1655 and 1531 cm^−1^, indicating the presence of BSA's amide I and II linkages. The presence of pure EVO within BNPs was confirmed by the prominent pure EVO peaks at 1446, 1245, and 735 cm^−1^ that were visible in ENPs. As a result, the observed FTIR peaks demonstrate that EVO was successfully entrapped within BNPs.

X-ray Diffraction (XRD), another efficient technique, was used for verifying the encapsulation of pure EVO in BNPs. As shown in Fig. 2b, pure EVO displayed sharp XRD patterns, which are distinctive patterns of pure EVO’s crystalline nature and corroborated by a previously reported study^26^, while the XRD diffraction patterns of ENPs have not shown any characteristic of pure EVO peak, which confirmed its amorphous state.

Differential Scanning Calorimetry (DSC) thermograms of EVO, BSA, BNPs and ENPs were represented in Fig. 2c. The endothermic peak was observed at ~ 294 °C for EVO which is the endothermic point of EVO, also found in a reported study^28^. As ENPs missed the EVO peak, demonstrating that the pure EVO crystal nature was lost and pure EVO encapsulated in BNPs. The TGA analysis was performed and data are shown in Fig. 2d. Pure EVO and pure BSA both lost up to 3% and 17% of their original weights at their melting points. Up to 25% of the total weight of the ENPs was lost after encapsulation of pure EVO. The increased total weight loss could be attributed to the organic chemical component (EVO) encapsulated inside the nanoparticles (BNPs).

### pH-dependent EVO release from ENPs

**Figure 3:**
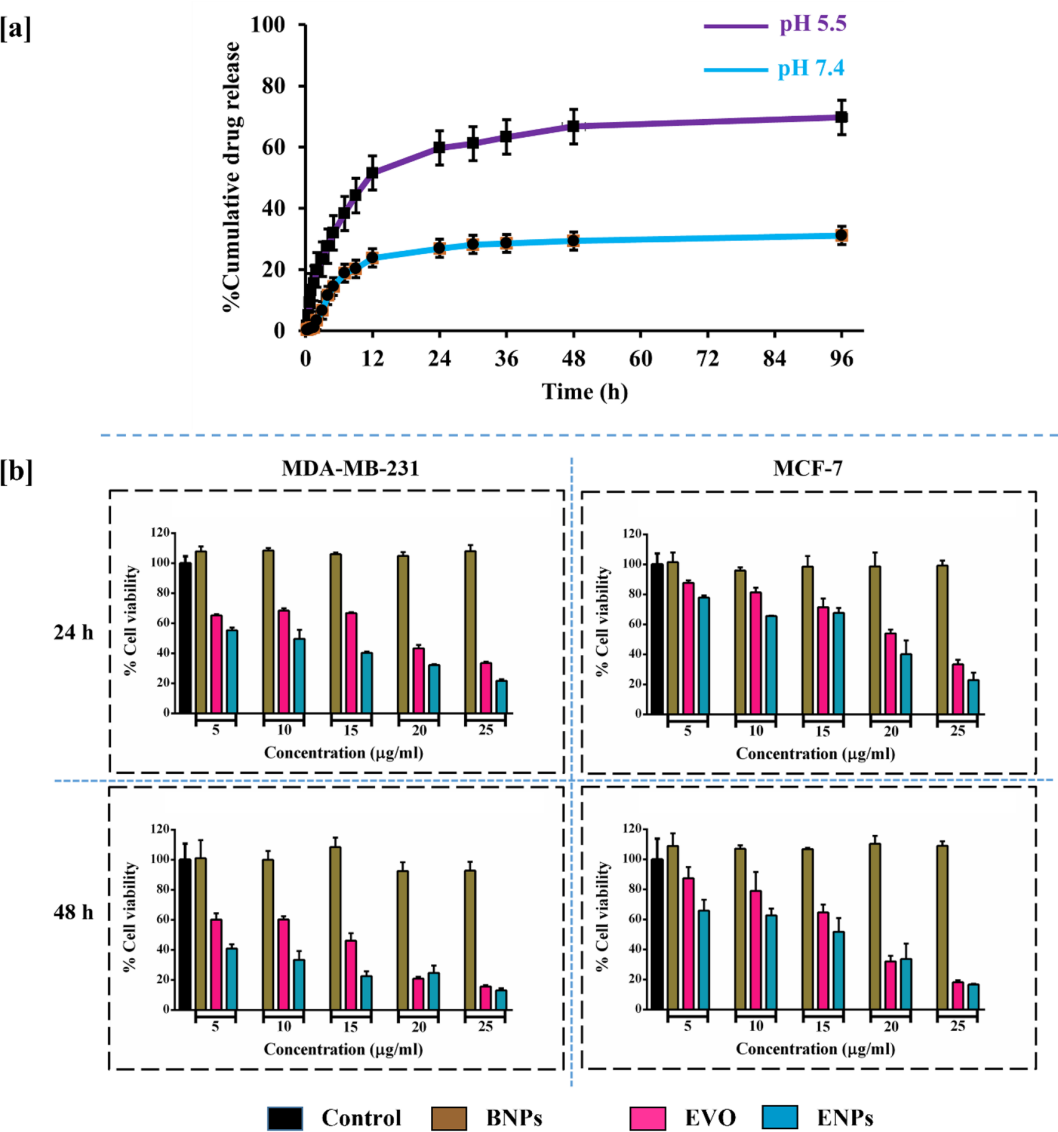
In-vitro drug release (**a**). Release study of EVO from ENPs was performed in two different pH conditions (pH 7.4 and 5.5). In-vitro cytotoxicity assay (**b**). Cytotoxic effects of BNPs, pure EVO and ENPs (5, 10, 15, 20 and 25 µg/ml) against human breast cancer cells for 24 and 48 h.

To investigate the pH-dependent EVO release from ENPs, drug release using the dialysis bag diffusion method was performed in two pH conditions particularly pH 7.4 and pH 5.5, which mimic the physiological pH state and the acidic environments of tumor/tissue, respectively. As shown in Fig. 3a, slow release of EVO from the ENPs was observed about 31% even after 96 h at pH 7.4; while in an acidic medium (pH 5.5), around 70% EVO was released after the same time interval. Drug release studies indicated that ENPs managed to release EVO in a pH-dependent way. The tumor microenvironment generally has acidic pH than healthy and normal tissues, thus, pH-dependent drug release from ENPs system could be useful for enhanced drug release and subsequent killing of cancer cells.

### In-vitro cytotoxicity and morphological analysis

Figure 3b illustrates the cytotoxicity of ENPs on human breast cancer cell lines. BNPs, EVO and ENPs with different concentrations were incubated with cancer cells for 24 and 48 h. The cell viability was observed more than 90% in the BNPs treated cells in both time-point (24 and 48 h). Obtained results with BNPs suggested that BNPs did not exert any cytotoxicity, hence they are biocompatible and non-toxic for breast cancer cells. Pure EVO and ENPs reduced the cancer cells viability in both cancer cell lines in a time- and dose-dependent manner. However, after treatment with ENPs, cell viability was reduced significantly compared to EVO, inferring that cytotoxicity of pure EVO is enhanced after encapsulation in BNPs, possibly due to increased uptake of pure EVO by breast cancer cells.

IC_50_ values were calculated for each treatment group against both cell lines and presented in Table 2. After treatment with pure EVO and ENPs against MDA-MB-231 cells, The IC_50_ values were calculated to be 17.48 and 7.86 µg/ml, respectively after 24 h, which were 9.47 and 3.44 µg/ml after treatment of 48 h. For MCF-7 cells IC_50_ results for pure EVO and ENPs were determined as 20.98 µg/ml and 15.36 µg/ml, respectively after 24 h whereas 15.46 µg/ml and 11.48 µg/ml at 48 h. Compared to EVO, IC_50_ values for ENPs were observed to almost double in both cell lines (Fig. S3). Thus, MTT assay results demonstrated that the cytotoxicity of ENPs against breast cancer cells was enhanced compared to pure Evo.

**Table 2:** IC_50_ values (µg/ml) of EVO and ENPs against MDA-MB-231 and MCF-7 cells.

Time	EVO (µg/ml)	ENPs
1. MDA-MB-231		
24 h	17.48	7.86
48 h	9.47	3.44
2. MCF-7		
24 h	20.98	15.36
48 h	15.46	11.48

In a reported study, the formation of apoptotic bodies, nuclear fragmentation, chromatin margination, cell shrinkage, and concentrated cytoplasm were all seen in EVO treated cells^29^. As shown in Fig. S4, apoptotic features like swelling and rounded morphology were observed in pure EVO and ENPs incubated cancer cells. However, compared to pure EVO, a decrease in cell number, cytoplasm shrinkage and cell vacuoles were observed higher. According to the findings, ENPs decreased cancer cell proliferation more effectively and caused more cell death than pure EVO.

### ENPs decrease the colony formation activity of breast cancer cells

The antiproliferative activity of ENPs was further assessed using a colony formation assay. As seen in Fig. S5, BNPs treated breast cancer cells showed almost similar number of colonies which suggested that BNPs had no significant inhibition on colony formation. EVO and ENPs significantly inhibited colony formation in breast cancer cells. However, the colony inhibition capacity was seen more in ENPs treated cancer cells. Colony formation assay results implied that ENPs have more anticancer activity and the ability to inhibit colony formation compared to pure EVO.

### ENPs enhance apoptosis in breast cancer cells

To assess the apoptotic changes induced by ENPs, Acridine Orange-Ethidium Bromide (AO-EtBr) dual staining method was used. The cells were incubated with ENPs and fluorescence analysis was done by fluorescence microscope. The dyes AO and EtBr are used to distinguish between necrotic, apoptotic, and normal cells^30^. AO dye penetrates and emits green fluorescence when bound to the DNA of normal and healthy cells with intact membranes. Whereas, EtBr dye stains dead cells and late apoptotic cells emitting orange-red fluorescence.

**Figure 4:**
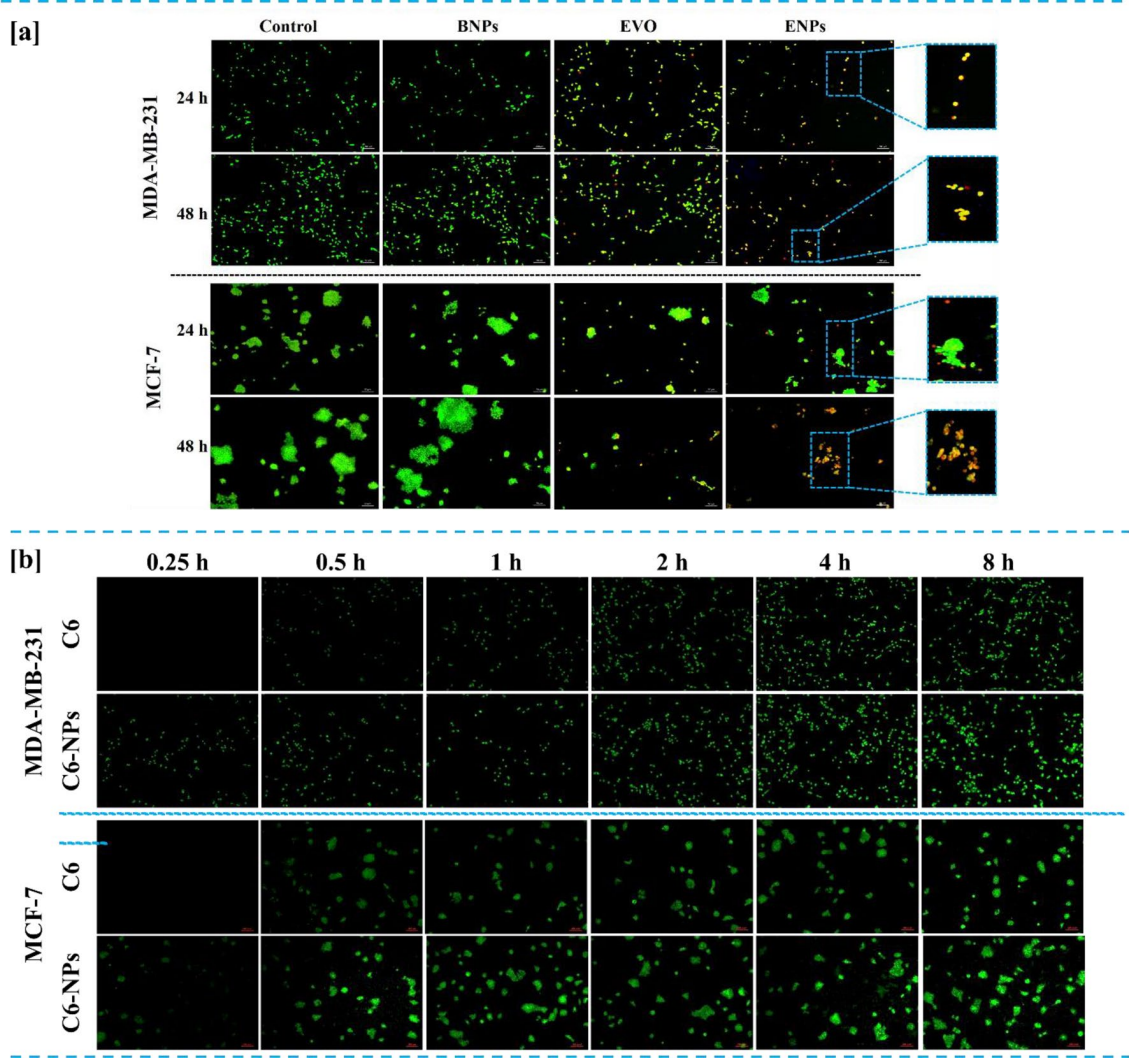
Detection of apoptosis by AO-EtBr staining assay (**a**). Cells were treated with BNPs, pure EVO and ENPs at a concentration of 5 µg/ml for 24 h and 48 h. The representative fluorescence microscopic images were captured (50 µm, n = 3). In-vitro cellular uptake study (**b**). Fluorescent images of cancer cells treated with pure Coumarin-6 (C-6) and C6-NPs for different time intervals (50 µm, n = 3).

BNPs treated cells were healthy as indicated by green fluorescence (Fig. 4a). However, in cancer cells treated with pure EVO and ENPs, cells displayed orange-red fluorescence indicating apoptotic and necrotic cells. Compared with pure EVO, ENPs manifested more potent apoptotic characteristics, which could be attributed to the high anticancer activity promoted by ENPs (Fig. S6).

### ENPs enhance EVO uptake by human breast cancer cells

To check the internalization of prepared ENPs by cancer cells, cellular uptake analysis was investigated. Coumarin 6 (C6) was used as probe dye and encapsulated in BNPs by using the same protocol used during the preparation of ENPs. Breast cancer cells were incubated with only C6 and C6-NPs and fluorescence images were captured using a fluorescence microscope. As displayed in Fig. 4b, the green fluorescence intensity was higher in C6-NPs treated cancer cells than in C6 alone. Albumin nano formulations offer improved cellular uptake compared to free drug. The small size and surface characteristics of nanoparticles facilitate their uptake in cancer cells via receptor mediated endocytosis, leading to enhanced internalization. Reported studies suggest that Albumin nanocarriers have high binding affinity towards gp-60 and SPARC receptors that are overexpressed in breast cancer cells^31^.

Cellular uptake results demonstrated that ENPs have a stronger ability to penetrate the cell membranes into the breast cancer cells suggesting that internalization and bioavailability of EVO could be significantly enhanced after encapsulation within BNPs.

### Cell cycle arrest induced by ENPs

The cell cycle protects the DNA and plays a vital role in the process of cell proliferation and growth^32^. It is well known that the G2/M checkpoint prevents the onset of mitosis in cells until DNA damage has been repaired. If the damage cannot be repaired, checkpoint signaling induces cells to undergo apoptosis, which results in cell death^33^. Thus, the effects of ENPs on cell cycle arrest were examined using flow cytometry.

**Figure 5:**
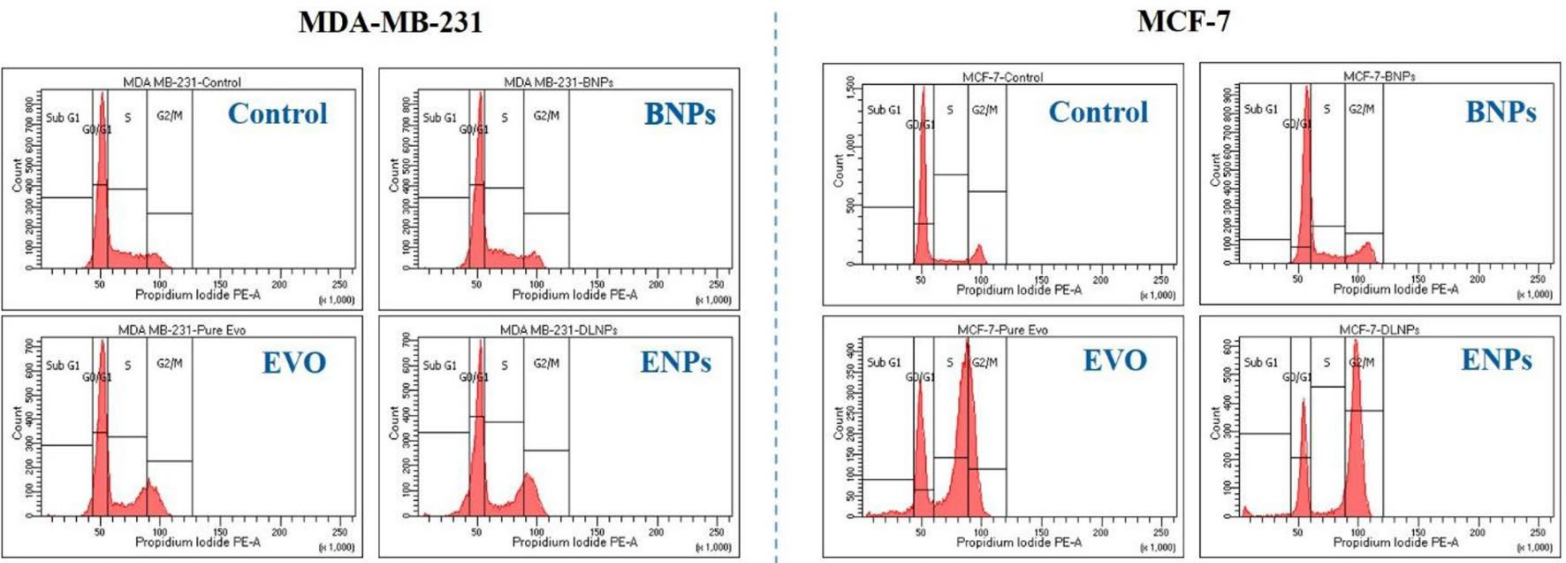
Cell cycle analysis. Breast cancer cells were treated with BNPs, pure EVO and ENPs for 24 h, stained with PI and cell cycle analysis was evaluated using flow cytometry.

Figure 5 showed the cell cycle arrest in Propidium Iodide (PI) stained breast cancer cells after incubation with 2.5 µg/ml of BNPs, EVO and ENPs for 24 h. No significant alterations in the phases of G0/G1, S, or G2/M were observed in BNPs treated cells. However, in ENPs treated cells, the percentage of cells in the G2/M phase was enhanced compared to EVO. In MDA-MB-231 cells, 15.5% and 19.7% cells were arrested in the G2/M phase after treatment with EVO and ENPs, respectively. While, in the MCF-7 cells, 23.6% and 62.0% cells were in the G2/M phase. The cell cycle arrest suggested that EVO arrests the cells in the G2/M phase but after encapsulating in BNPs, the uptake efficiency in the cells was much higher as compared to the pure drug and thus, cells were arrested in the G2/M phase was higher in ENPs.

### Effect of ENPs on mRNA expression of apoptosis markers

**Figure 6:**
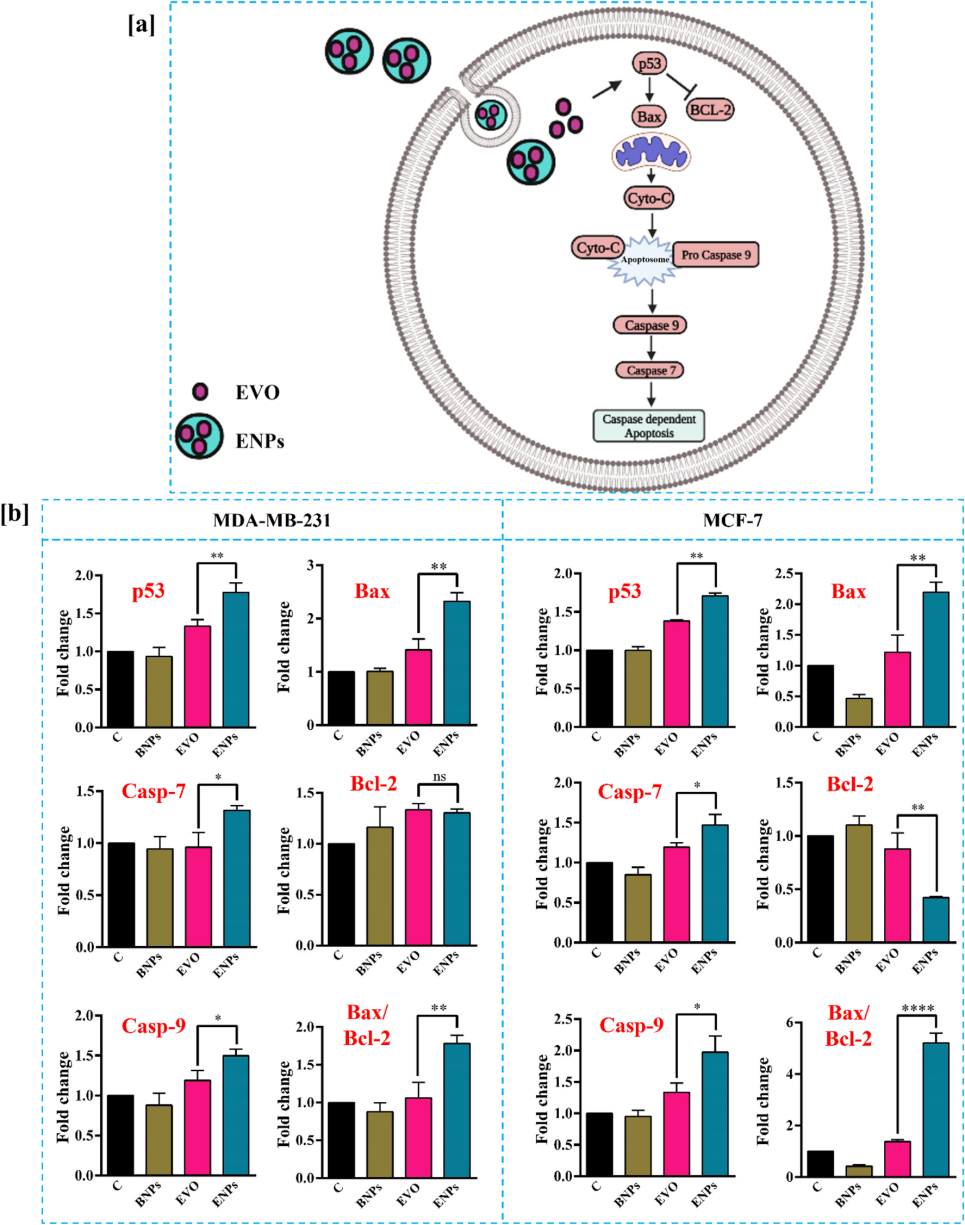
Schematic diagram of the anticancer mechanism of prepared ENPs via p53, Bax/Bcl-2 and caspase pathway (**a**). Quantitative gene expression analysis (**b**) of p53, Caspase-7, Caspase-9, Bax, Bcl-2 and Bax/Bcl-2 ratio using real-time RT-PCR. Breast cancer cells were incubated with BNPs, EVO and ENPs for 24 h and qRT-PCR was performed. mRNA was normalized with GAPDH. Values are shown in mean ± S.D. of n = 3 (*P < 0.05, **P < 0.01, ***P < 0.001 and ****p < 0.0001).

To determine the mechanism of apoptosis induced by ENPs, the relative gene and protein expressions were examined. Apoptosis is tightly controlled by pro- and anti-apoptotic proteins such as p53, Bax, Bcl-2, and caspases (Fig. 6a). p53 can activate pro-apoptotic molecules such as Bax, Bak and increase mitochondrial permeability and release cytochrome C. Cytochrome C release initiates the development of apoptosome complex and leads to initiation of further caspase cascade activation including caspase-7 and caspase-9 and ultimately induce apoptosis-mediated cell death^33^. Bax overexpression accelerates cell death while Bcl-2 overexpression improves cell survival by decreasing apoptosis^34^. The ratio of Bax to Bcl-2 proteins within a cell is critical for determining the cell's susceptibility to apoptosis. When the ratio of Bax to Bcl-2 is high, it favors apoptosis, as there is more pro-apoptotic Bax than anti-apoptotic Bcl-2. In contrast, a low Bax to Bcl-2 ratio inhibits apoptosis, as there is a higher proportion of anti-apoptotic Bcl-2^35^. Thus, in this study, we have examined the mRNA expression of p53, Bax, Bcl-2, caspase-7 and caspase-9 using qRT-PCR. Bax/Bcl-2 ratio was also measured. GAPDH expression was chosen as the endogenous control gene.

As demonstrated in Fig. 6b, the difference in gene expression between the cells treated with BNPs and the control groups was not significant. Further, the qRT-PCR assay revealed that EVO and ENPs induced the expression of p53, caspase-7, caspase-9, and Bax and reduced the expression of Bcl-2. However, compared to EVO, ENPs significantly increased the expression of p53 (**p < 0.01), caspase-7 (*p < 0.05), caspase-9 (*p < 0.05), Bax (**p < 0.01) and reduced mRNA level of Bcl-2 in MDA-MB-231 cells.

Almost the same gene expression pattern was observed in all the genes including p53 (**p < 0.01), caspase-7 (*p < 0.05), caspase-9 (*p < 0.05), Bax (**p < 0.01) and Bcl-2 (**p < 0.01) in MCF-7 treated cells. ENPs induced ratio of Bax/Bcl-2 in both MDA-MB-231 (**p < 0.01) and MCF-7 cells (****p < 0.0001) compared to EVO. Overall, qRT-PCR results demonstrated that human breast cancer cells underwent apoptosis induced by ENPs through the caspase-dependent Bax/Bcl-2 pathway.

### Western blotting

The apoptotic protein expression including p53, Bax and Bcl-2 was analyzed using western blotting analysis. Western blotting results were found consistent with the mRNA expression study.

**Figure 7:**
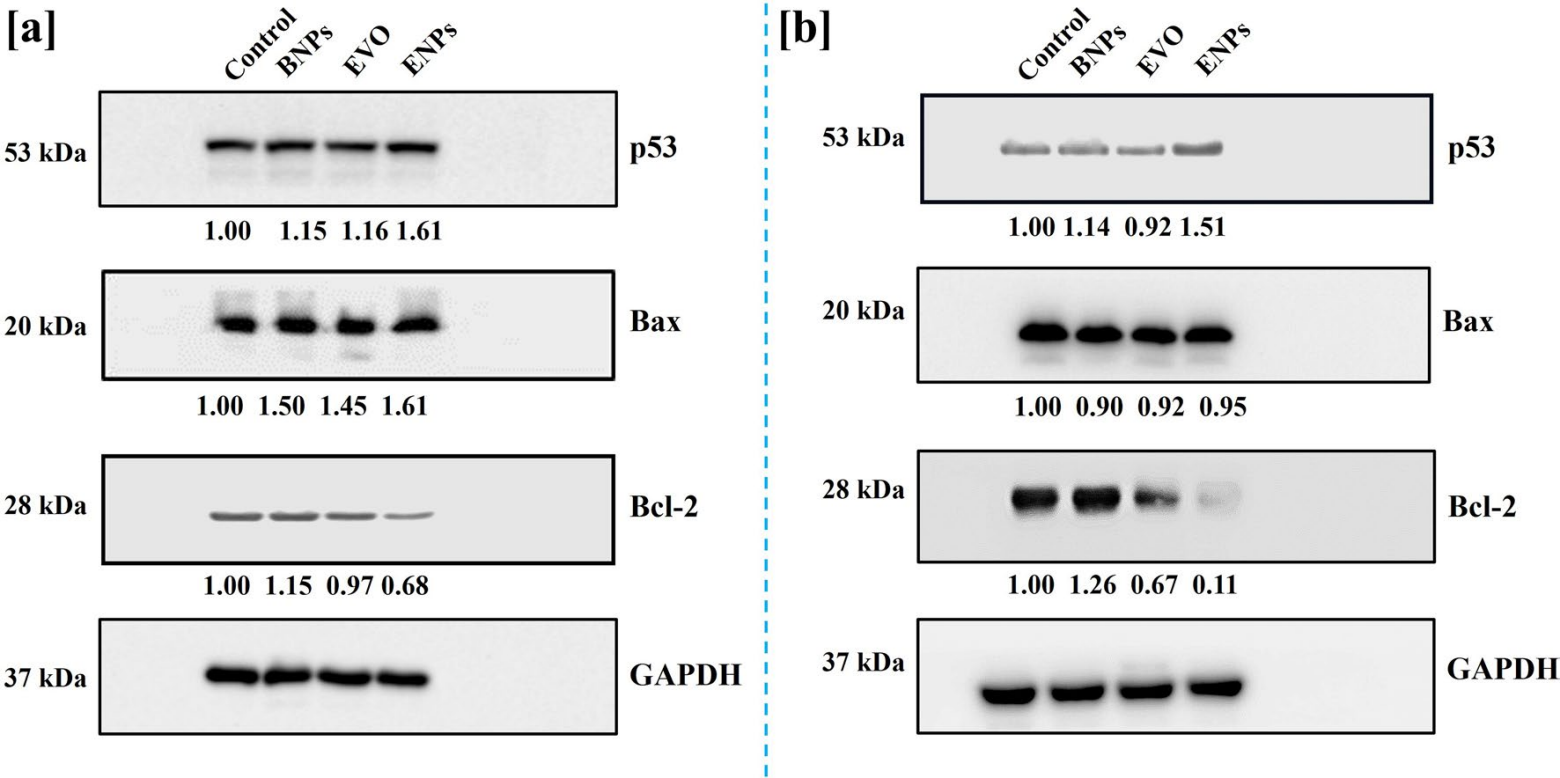
Western blot analysis. The protein expression changes of p53, Bax and Bcl-2 in MDA-MB-231 cells (**a**) and MCF-7 cells (**b**) after treatment with BNPs, EVO, and ENPs for 24 h.

As shown in Fig. 7, BNPs slightly altered protein expression in both cell lines which was non-significant, whereas after treatment with EVO and ENPs, the p53 expression was increased in both cell lines. However, the cells treated with ENPs showed a significantly enhanced expression of p53 as compared to EVO (Fig. S8 and Fig. S9).

As discussed in the mRNA expression study, Bax/Bcl-2 ratio acts as a regulator of the apoptosis mechanism in the cells and thus, we also assessed Bax/Bcl-2 protein ratio^36^. Bax expression was significantly increased and Bcl-2 was reduced by ENPs compared to EVO. Thus, Bax/Bcl-2 ratio was observed higher in ENPs treated breast cancer cells (Fig. S10). These findings showed that ENPs increased the expression of p53 and the ratio of Bax/Bcl-2 was higher in ENPs group as compared to the EVO suggesting ENPs induced apoptosis in human breast cancer cells via p53 and Bax/Bcl-2 mediated pathways. Our finding is also in line with the previously reported literature demonstrating the anticancer activity of EVO-mediated through p53 and Bax/Bcl-2 pathways^37^.

## Experimental section

### Materials

EVO (> 99%), BSA (> 99), 8% Glutaraldehyde, MTT, C6, TRIZOL, DMSO, AO, EtBr and PI were procured from Sigma (Saint Louis, MO, USA). Thermo-fisher Scientific (Waltham, MA, USA) supplied Dulbecco's modified essential medium with high glucose (DMEM-HG), Minimum Essential Medium (MEM), Fetal Bovine Serum (FBS), and 0.25% Trypsin EDTA. Antibodies against GAPDH, p53, Bax and Bcl-2 were procured from Cell Signalling Technology (CST) (Denver, CO, USA). Secondary anti-rabbit and anti-mouse antibodies, cDNA synthesis kit, and SYBR green were procured from Bio-Rad (Hercules, CA, USA).

### Preparation and purification of BNPs and ENPs

BNPs and ENPs were synthesized using the desolvation method^38^. In brief, for the preparation of BNPs, BSA (100 mg/ml concentration) was dissolved in ultrapure water and the pH was increased to 8.5 by adding NaOH. Then, BSA NPs were formed by dropwise (1 ml/min) addition of 8 ml of ethanol as a desolvating agent, under constant stirring at RT till the milky appearance. 0.23 ml glutaraldehyde was added for cross-linking purpose and the reaction was kept on continuous stirring for 24 h. The next day, unbound BSA and an excess amount of glutaraldehyde were discarded by three repetitive centrifugation and the pellet was dispersed in ultrapure water. The prepared nanoparticles were stored at − 80 °C for 24 h and lyophilized using lyophilizer (Labconco) for further characterization.

For the preparation of ENPs same protocol was followed as mentioned for BNPs. In brief, 20 mg EVO was dissolved in 8 ml ethanol. The EVO solution was then added to BSA solution dropwise at a flow rate of 1 ml/min. The conditions and the rest of the steps were followed as per the BNPs preparation protocol.

### Physicochemical characterization of BNPs and ENPs

The particle size, PDI and ZP of the BNPs and ENPs were analyzed using DLS (Zetasizer, NanoS90, Malvern, U.K.). The samples were dispersed in ultrapure water and 1 ml sample was analyzed at 25 °C using DLS. The morphology and surface of the prepared BNPs and ENPs were examined using FE-SEM (Carl Zeiss, JEOL JSM-7600F) and TEM (JEOL JEM-2100, Tokyo, Japan) analysis.

Encapsulation of EVO within BNPs was confirmed using FTIR (Nicolet iS5, Thermo Scientific, USA) and XRD (D8 Advance, Bruker, Germany) analysis. Samples were scanned in the ranges of 4000–400 cm^−1^ for the FTIR analysis. XRD patterns were scanned in the 2θ range between 5° to 50°. 40 kV operating voltage and 30 mA operating current were applied for the sample analysis.

Thermodynamic characteristics of BNPs and ENPs were assessed using DSC (DSC 4000, PerkinElmer, MA, U.S.A) and TGA (TG/DTA 7300, Exstar) analysis. For DSC analysis samples were sealed in standard aluminium pans and then scanned in the range of 30 to 350 °C. An empty aluminium pan was for reference. TGA analysis was carried out to investigate the thermal stability of the BNPs and ENPs. For that samples were scanned in the 30 °C to 700 °C temperature range.

### Measurement of %EE and %LC

To know the %EE and %LC, ENPs were dispersed in ultrapure water and centrifuged at 10,000 rpm for 10 min at Room Temperature (RT) and in dark conditions. EVO concentration was measured using UV–Vis spectroscopy (Evolution 201 UV–visible spectrophotometer, Thermo scientific) and calculated as follows:

$$ \begin{aligned}   {\text{\% EE}}~ &  = \frac{{{\text{Initial }} {\text{concentration }}{\text{of }}{\text{EVO}} - {\text{EVO}}\,{\text{concentration}}\,{\text{in}}\,{\text{supernatant}}}}{{{\text{Initial}}\,{\text{concentration}}\,{\text{of}}\,{\text{EVO}}}}~~ \times 100 \\    {\text{\% LC}}~ &  = \frac{{{\text{Initial}}\,{\text{concentration}}\,{\text{of}}\,{\text{EVO}} - {\text{EVO}}\,{\text{concentration}}\,{\text{in}}\,{\text{supernatant}}}}{{{\text{Initial}}\,{\text{concentration}}\,{\text{of}}\,{\text{EVO}}\,{\text{and}}\,{\text{BSA}}}}~~ \times 100 \\  \end{aligned}  $$

### EVO release profile from ENPs

EVO release from prepared ENPs was determined using the dialysis bag diffusion method. The release study was carried out in acidic medium (pH 5.5) and physiological medium (pH 7.4) mimicking tumor microenvironment and physiological condition. For the drug release study, ENPs (2 mg/ml) were dispersed and poured in a dialysis bag (M.W. 12–14 kDa). Dialysis bag submerged in 50 ml of respective medium in RT with 100 rpm. After a definitive time, 2 ml of medium was taken for analysis and replaced with fresh buffer. %Cumulative drug release was estimated for 96 h using UV–Vis spectroscopy at 225 nm.

### Cell culture

The Human breast cancer MDA-MB-231 and MCF-7 cells were purchased from National Centre for Cell Science (NCCS, Pune, India). Cells were cultured in DMEM-HG (MDA-MB-231) and MEM (MCF-7) media containing 10% FBS and 37 °C temperature provided with 5% CO_2_. After 2–3 days, the medium was replaced with a fresh one. Cells based experiments were performed after reaching 80% to 90% confluency.

### Cytotoxicity assessment by MTT assay

In-vitro anticancer activity of prepared ENPs on breast cancer cells was evaluated using MTT assay. For this, cells were grown in 96-well culture plates at 7000 cells per well density. Further, cells were incubated with various concentrations (5–25 µg/ml) of BNPs, EVO and ENPs for 24 h and 48 h. Then, media from each well was discarded and freshly prepared 100 µL MTT reagent was added. 100 µL of DMSO was replaced with MTT reagent and kept for a further 15 min. Finally, Optical Density (O.D.) at 570 nm was measured by using a multimode plate reader and percentage cell viability was calculated.

The morphological behavior of cells after treatment with BNPs, EVO and ENPs at 10 µg/ml concentration was observed and photographs were captured.

### Colony formation assay

Anticancer and colony inhibition efficacy of ENPs was examined by performing a colony formation assay^39^. For this 800 cells were cultured in 6-well culture plates. Then, cells were incubated with BNPs, EVO and ENPs at 10 µg/ml concentration for 7 days. After one week, images were captured using a digital camera followed by crystal violet staining.

### AO-EtBr staining assay for apoptosis detection

The AO-EtBr staining method was used to determine the apoptotic changes induced by ENPs in breast cancer cells as per previously reported^40^. Cells were seeded and next day treated with 10 µg/ml of BNPs, EVO and ENPs for 24 h and 48 h time periods. Then, cells were stained with dual dye (AO-EtBr) for 2 min followed by PBS washing. Microscopic images were captured using a fluorescence microscope.

### Assessment of ENPs internalization by breast cancer cells

To confirm the uptake of ENPs by breast cancer cells, a cellular uptake study was performed. For that, C6 was encapsulated in BNPs using the same method used in the preparation of ENPs. Then, cells were seeded and the next day treated with C6 and C6 NPs at 100 ng/ml concentration. After a definite time, microscopic images were captured by a fluorescence microscope.

### Detection of cell cycle arrestment by FACS analysis

For the cell cycle analysis, cancer cells were cultured and incubated with EVO, BNPs and ENPs for 24 h. The next day cells were trypsinized, washed and stained with PI dye for 15 min and the cell cycle was performed using FACS Aria III (BD Biosciences, USA).

### RNA Isolation and mRNA expression study

To assess the apoptosis mechanism of ENPs at the molecular level, the expression of target genes (p53, Bax, Bcl-2, caspase-7 and caspase-9) was evaluated by qRT-PCR. For that, cells were grown and treated with 2.5 µg/ml of EVO, BNPs and ENPs and total RNA was isolated using the TRIzol method. The iScript cDNA synthesis kit (Bio-Rad, Hercules, CA, USA) was used to perform reverse transcription from the total RNA (500 ng) of samples and cDNA synthesized. The final reaction for qRT-PCR (10 µl) contained 5 µl of SYBR green master mix, 2 µl cDNA (300 ng per reaction), 0.2 µl forward and 0.2 µl reverse primer and Milli-Q water. Then, the expression of target genes (A list of primer sequences was provided in Table S1) was examined using the applied biosystem 7500 Real-Time PCR system. In order to measure the level of mRNA expression, the 2 delta-delta CT technique was used, and the results were normalized against the housekeeping gene GAPDH. To verify that results could be reliably reproduced, each reaction was carried out three times.

### Western blotting

The apoptosis mechanism was also explored at the protein expression level using western blotting. The cells were lysed in a lysis buffer (the cocktail of phosphatase and protease) and lysates were collected. Protein concentration from all groups was equalized by the Bradford method. The proteins were separated using SDS-PAGE and transferred to PVDF membranes. The membranes were stained with ponceau stain and cut horizontally according to the expected molecular weight of the protein to be probed by western blotting (Fig. S7). The horizontally cut membrane strips were then blocked by incubation with 5% non-fat dry skim milk powder. The membrane strips were probed overnight at 4 °C with p53, Bax, Bcl-2, and GAPDH specific primary antibodies. The membrane strips were washed twice in wash buffer before incubating with secondary antibodies for 2 h at RT. The ECL method was used to detect protein bands and images were captured by Bio-Rad Chemi Doc (Hercules, CA, USA). Densitometrical analysis was performed using ImageJ software (NIH, Maryland, USA).

### Statistical analysis

All experiments were done three times and obtained results were represented as mean ± standard deviation and p < 0.05 value considered as statistically significant during result analysis.

## Conclusion

In this study, we have prepared ENPs using the desolvation method and characterized by different physicochemical techniques including DLS, FESEM, FTIR, XRD, DSC and TGA analysis. *In-vitro* drug release study suggested the sustained drug release profile. *In-vitro* cytotoxicity assay demonstrated that ENPs showed enhanced anticancer activity against breast carcinoma cells as compared to the pure drug. Furthermore, fluorescence microscopic studies represented the enhanced cellular uptake of ENPs, which inhibits colony formation and induces apoptosis to greater extent as compared to pure EVO. As a result of increased cellular uptake, stability and sustained drug release, the intracellular concentration of EVO was enhanced at the target site, resulting in enhanced cytotoxicity, decreased colony formation and enhanced apoptosis through ENPs in MDA-MB-231 and MCF-7 cells. ENPs arrested the cells in the G2/M phase more efficiently and led to apoptosis. The mRNA expression study and western blotting results demonstrated upregulation of p53 and Bax expression and downregulation of Bcl-2 expression. Thus, molecular findings suggested that ENPs induced cytotoxicity and apoptosis via p53 and Bax/Bcl-2 pathways. Altogether, physicochemical characterizations, *in-vitro* anticancer studies, mRNA and protein expression studies demonstrated that ENPs could be a potential strategy to combat breast cancer.

### Supplementary Information

Supplementary Information.

## Data Availability

All experimental data generated during this study are included in this manuscript and supplementary information files.

## References

[1] Sung H (2021). Global cancer statistics 2020: GLOBOCAN estimates of incidence and mortality worldwide for 36 cancers in 185 countries. CA Cancer J. Clin..

[2] Arnold M (2022). Current and future burden of breast cancer: Global statistics for 2020 and 2040. The Breast.

[3] Yadav P, Ambudkar SV, Rajendra Prasad N (2022). Emerging nanotechnology-based therapeutics to combat multidrug-resistant cancer. J. Nanobiotechnol..

[4] Choudhari AS, Mandave PC, Deshpande M, Ranjekar P, Prakash O (2020). Phytochemicals in cancer treatment: From preclinical studies to clinical practice. Front. Pharmacol..

[5] Sun Q, Xie L, Song J, Li X (2020). Evodiamine: A review of its pharmacology, toxicity, pharmacokinetics and preparation researches. J. Ethnopharmacol..

[6] Panda M, Tripathi SK, Zengin G, Biswal BK (2023). Evodiamine as an anticancer agent: A comprehensive review on its therapeutic application, pharmacokinetic, toxicity, and metabolism in various cancers. Cell Biol. Toxicol..

[7] Li C-G (2019). Evodiamine augments NLRP3 inflammasome activation and anti-bacterial responses through inducing α-tubulin acetylation. Front. Pharmacol..

[8] Pang S (2023). Discovery of an evodiamine derivative for PI3K/AKT/GSK3β pathway activation and AD pathology improvement in mouse models. Front. Mol. Neurosci..

[9] Wu P, Chen Y (2019). Evodiamine ameliorates paclitaxel-induced neuropathic pain by inhibiting inflammation and maintaining mitochondrial anti-oxidant functions. Hum. Cell.

[10] Hou X (2021). Evodiamine lowers blood lipids by up-regulating the PPARγ/ABCG1 pathway in high-fat-diet-fed mice. J. Nat. Prod..

[11] Jiang, M. et al. *Antidepressant-Like Effect of Evodiamine on Chronic Unpredictable Mild Stress Rats* (Elsevier, 2014). 10.1016/j.neulet.2014.12.038. 10.1016/j.neulet.2014.12.038 25545553

[12] Zha Y (2023). Dietary evodiamine inhibits atherosclerosis-associated changes in vascular smooth muscle cells. Int. J. Mol. Sci..

[13] Yuan X-L (2017). Cytological assessments and transcriptome profiling demonstrate that evodiamine inhibits growth and induces apoptosis in a renal carcinoma cell line. Sci. Rep..

[14] Chien C-C (2014). Activation of JNK contributes to evodiamine-induced apoptosis and G2/M arrest in human colorectal carcinoma cells: A structure-activity study of evodiamine. PLoS ONE.

[15] Wang Y, Ma H, Narula A, Liu L, Ahn KS (2022). Molecular targets and anticancer potential of evodiamine. Phytochem. Lett..

[16] Zhong Z-F, Tan W, Wang S-P, Qiang W-A, Wang Y-T (2015). Anti-proliferative activity and cell cycle arrest induced by evodiamine on paclitaxel-sensitive and-resistant human ovarian cancer cells. Sci. Rep..

[17] Bai X, Meng H, Ma L, Guo A (2015). Inhibitory effects of evodiamine on human osteosarcoma cell proliferation and apoptosis. Oncol. Lett..

[18] Li C (2019). Development of EGFR-targeted evodiamine nanoparticles for the treatment of colorectal cancer. Biomater. Sci..

[19] Karimi M (2016). Albumin nanostructures as advanced drug delivery systems. Expert Opin. Drug. Deliv..

[20] Hoogenboezem EN, Duvall CL (2018). Harnessing albumin as a carrier for cancer therapies. Adv. Drug Deliv. Rev..

[21] Solanki R, Rostamabadi H, Patel S, Jafari SM (2021). Anticancer nano-delivery systems based on bovine serum albumin nanoparticles: A critical review. Int. J. Biol. Macromol..

[22] Solanki R, Jodha B, Prabina KE, Aggarwal N, Patel S (2022). Recent advances in phytochemical based nano-drug delivery systems to combat breast cancer: A review. J. Drug Deliv. Sci. Technol..

[23] Visentini FF, Perez AA, Santiago LG (2022). Bioactive compounds: Application of albumin nanocarriers as delivery systems. Crit. Rev. Food. Sci. Nutr..

[24] Mittal A, Gandhi S, Roy I (2022). Mechanistic interaction studies of synthesized ZIF-8 nanoparticles with bovine serum albumin using spectroscopic and molecular docking approaches. Sci. Rep..

[25] Mitchell MJ (2021). Engineering precision nanoparticles for drug delivery. Nat. Rev. Drug. Discov..

[26] Qiu C, Gao L-N, Yan K, Cui Y-L, Zhang Y (2016). A promising antitumor activity of evodiamine incorporated in hydroxypropyl-β-cyclodextrin: Pro-apoptotic activity in human hepatoma HepG2 cells. Chem. Cent. J..

[27] Solanki R, Saini M, Mochi J, Pappachan A, Patel S (2023). Synthesis, characterization, in-silico and in-vitro anticancer studies of Plumbagin encapsulated albumin nanoparticles for breast cancer treatment. J. Drug. Deliv. Sci. Technol..

[28] Zou L (2016). Preparation, characterization, and anticancer efficacy of evodiamine-loaded PLGA nanoparticles. Drug Deliv..

[29] Shen H (2015). Evodiamine inhibits proliferation and induces apoptosis in gastric cancer cells. Oncol. Lett..

[30] Solanki R, Jangid AK, Jadav M, Kulhari H, Patel S (2023). Folate functionalized and evodiamine-loaded pluronic nanomicelles for augmented cervical cancer cell killing. Macromol. Biosci..

[31] Kratz F (2014). A clinical update of using albumin as a drug vehicle—A commentary. J. Control. Release.

[32] Barnum KJ, O’Connell MJ (2014). Cell cycle regulation by checkpoints. Cell Cycle Control Mech. Protoc..

[33] Senturk, E. & Manfredi, J. J. p53 and cell cycle effects after DNA damage. In *p53 Protocols* 49–61 (2013). 10.1007/978-1-62703-236-0_4 PMC4712920 23150436

[34] Hemann MT, Lowe SW (2006). The p53–Bcl-2 connection. Cell Death Differ..

[35] Raisova M (2001). The Bax/Bcl-2 ratio determines the susceptibility of human melanoma cells to CD95/Fas-mediated apoptosis. J. Investig. Dermatol..

[36] Shamas-Din A, Kale J, Leber B, Andrews DW (2013). Mechanisms of action of Bcl-2 family proteins. Cold Spring Harb. Perspect. Biol..

[37] Hu X (2018). Antiproliferative effects of alkaloid evodiamine and its derivatives. Int. J. Mol. Sci..

[38] Solanki R, Patel K, Patel S (2021). Bovine serum albumin nanoparticles for the efficient delivery of berberine: Preparation, characterization and in vitro biological studies. Colloids Surf. A Physicochem. Eng. Asp..

[39] Jangid AK (2023). Phenylboronic acid conjugated PAMAM G4 dendrimers augmented usnic acid delivery to gastric cancer cells. Eur. Polym. J..

[40] Solanki R, Patel S (2023). Preparation, characterization and in vitro anticancer efficacy of biotin-conjugated, silibinin loaded bovine serum albumin nanoparticles. Food Biosci..

