# Author Correction: Enhancing apoptosis-mediated anticancer activity of evodiamine through protein-based nanoparticles in breast cancer cells

**DOI:** 10.1038/s41598-024-56762-3

**Published:** 2024-03-14

**Authors:** Raghu Solanki, Pradeep Kumar Rajput, Bhavana Jodha, Umesh C. S. Yadav, Sunita Patel

**Affiliations:** 1 School of Life Sciences, Central University of Gujarat, Gandhinagar, 382030 India; 2 Special Centre for Medicine and Special Centre for Systems Medicine, Jawaharlal Nehru University, New Delhi, 110067 India

Correction to: *Scientific Reports* 10.1038/s41598-024-51970-3, published online 31 January 2024

The original version of this Article contained errors in Figure 7 and the Supplementary Figures 7, 8 and 9 where the molecular weight of Bax and the molecular weight of Bcl-2 were incorrect. The correct molecular weight of Bax is 20 kDa and molecular weight of Bcl-2 is 28 kDa.

The original Figure 7, accompanying legend and Supplementary Information file appear below.

**Figure 7:**
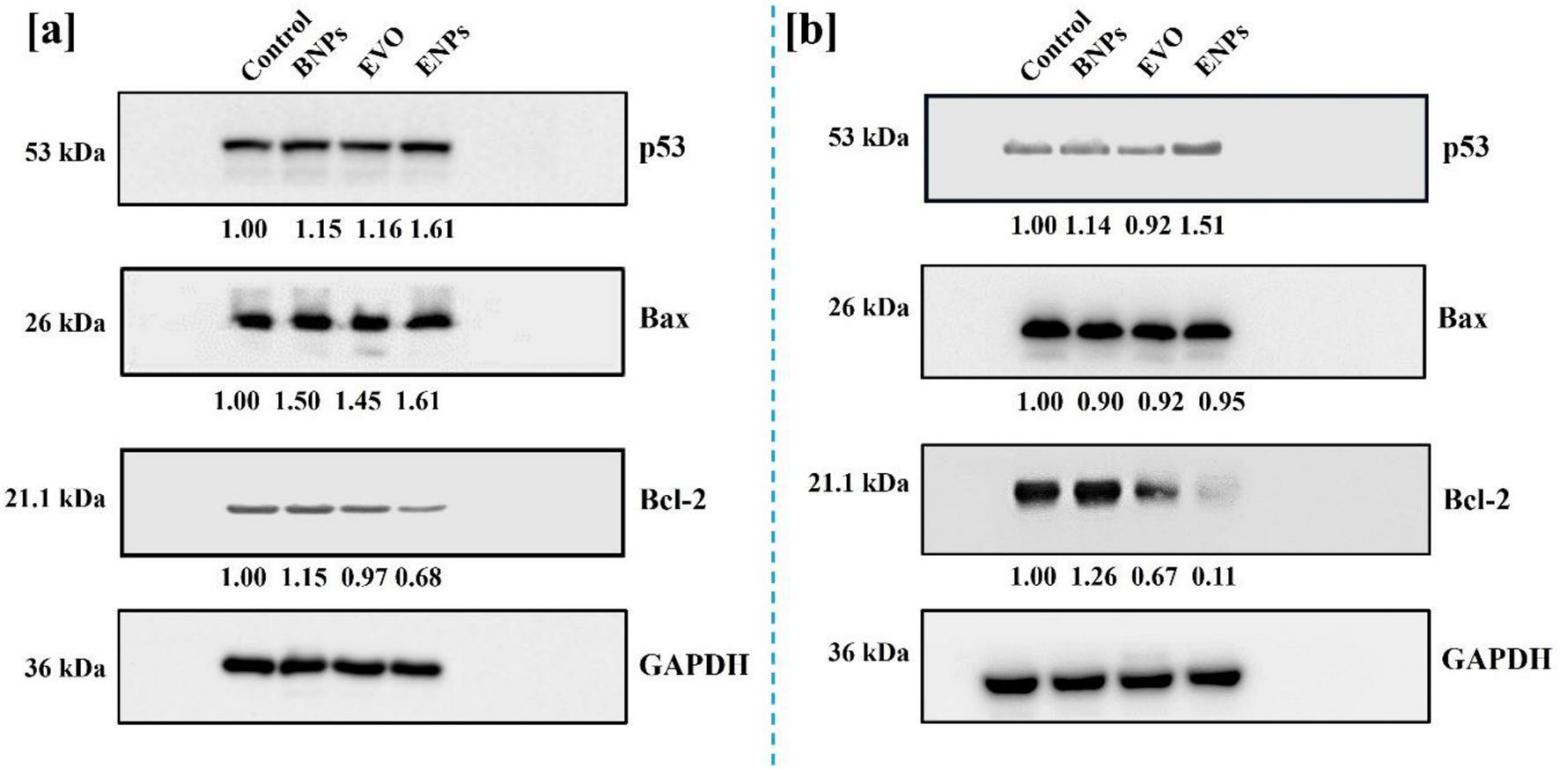
The caption to be typeset alongside it: Western blot analysis. The protein expression changes of p53, Bax and Bcl-2 in MDA-MB-231 cells (**a**) and MCF-7 cells (**b**) after treatment with BNPs, EVO, and ENPs for 24 h.

These errors have now been corrected in the original Article and in the Supplementary Information 1 file that accompanies the original Article.

### Supplementary Information

Supplementary Information.

