# Supplementary material for: Author Correction: Enhancing apoptosis-mediated anticancer activity of evodiamine through protein-based nanoparticles in breast cancer cells
Source: Sci Rep. 2024 Mar 14;14:6213. doi: 10.1038/s41598-024-56762-3 (PMC10940692; doi:10.1038/s41598-024-56762-3)
Supplement: Supplementary file 1 — Supplementary Information. [file 41598_2024_56762_MOESM1_ESM.docx]

Supplementary files

**Enhancing Apoptosis-Mediated Anticancer Activity of Evodiamine Through Protein-Based Nanoparticles in Breast Cancer Cells**

Raghu Solanki^1^, Pradeep Kumar Rajput^1^, Bhavana Jodha^1^, Umesh C.S. Yadav^2^, Sunita Patel^1*^

^1^ School of Life Sciences, Central University of Gujarat, Gandhinagar-382030, India

^2^ Special Centre for Medicine and Special Centre for Systems Medicine, Jawaharlal Nehru University, New Delhi – 110067, India

***Correspondence:**

Dr. Sunita Patel

Assistant professor

School of Life Sciences

Central University of Gujarat

Gandhinagar-382030, India

E-mail: [sunitap@cug.ac.in](mailto:sunitap@cug.ac.in)


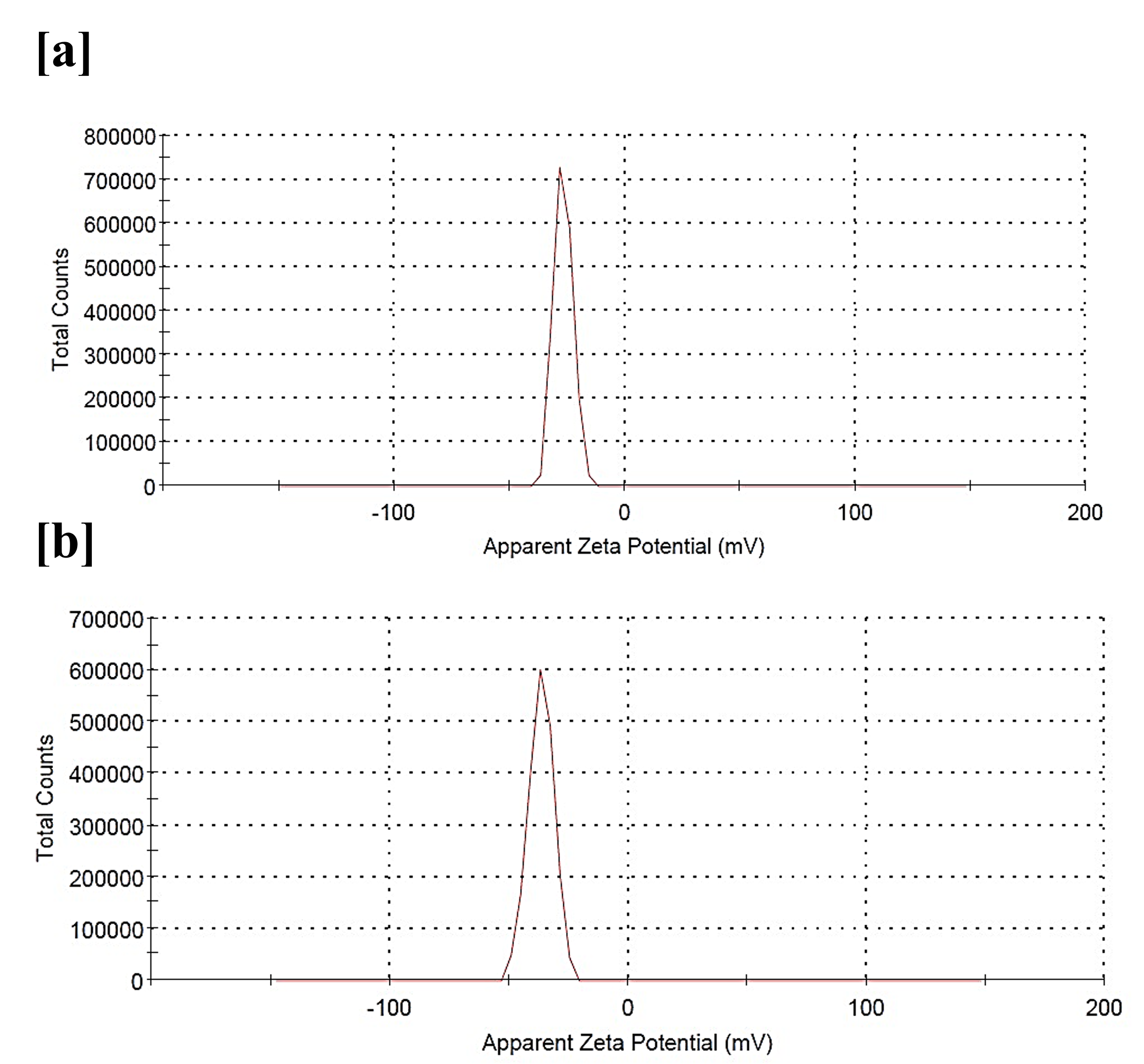


***Figure S1.*** *Zeta potential of BNPs (a) and ENPs (b) determined using DLS instrument.*


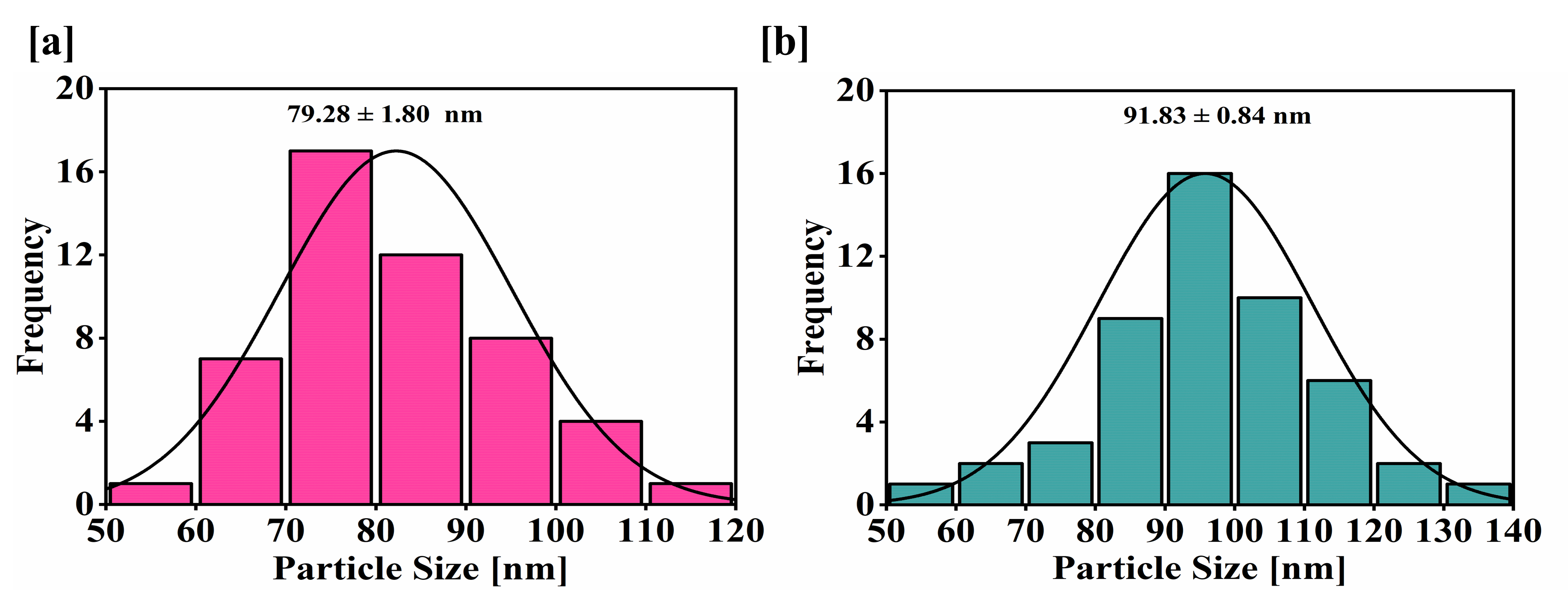


**Figure S2.** Size distribution curve graph of BNPs (a) and ENPs (b) plotted using ImageJ after FE-SEM analysis.


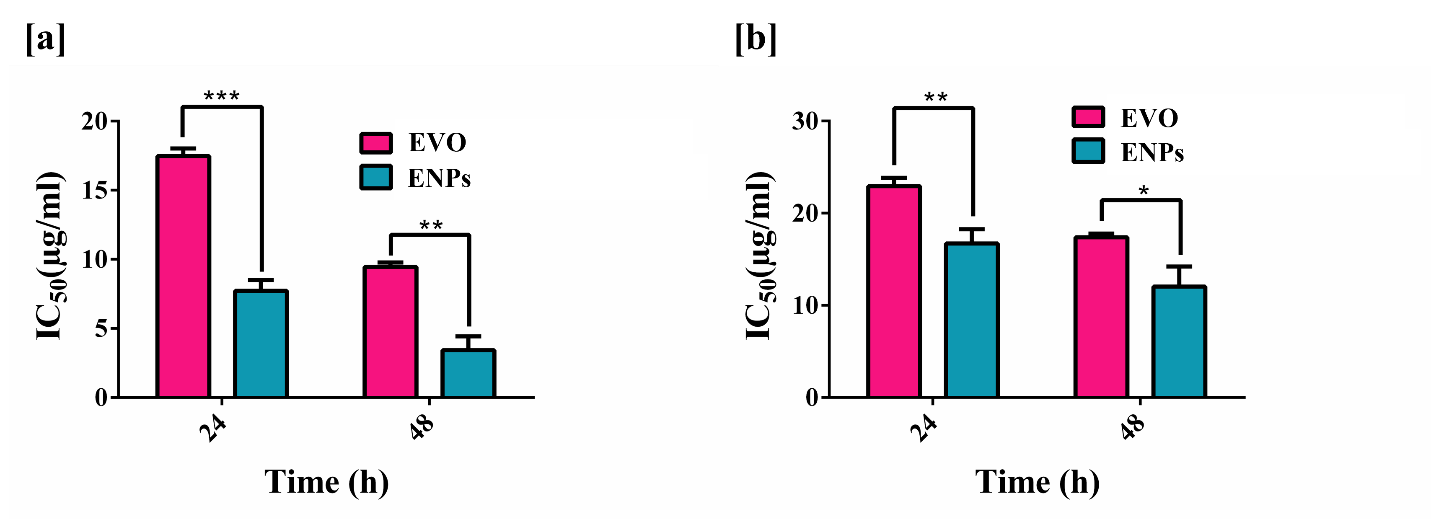


**Figure S3.** IC_50_ values of EVO and ENPs against MDA-MB-231 cells (a) and MCF-7 cells (b).


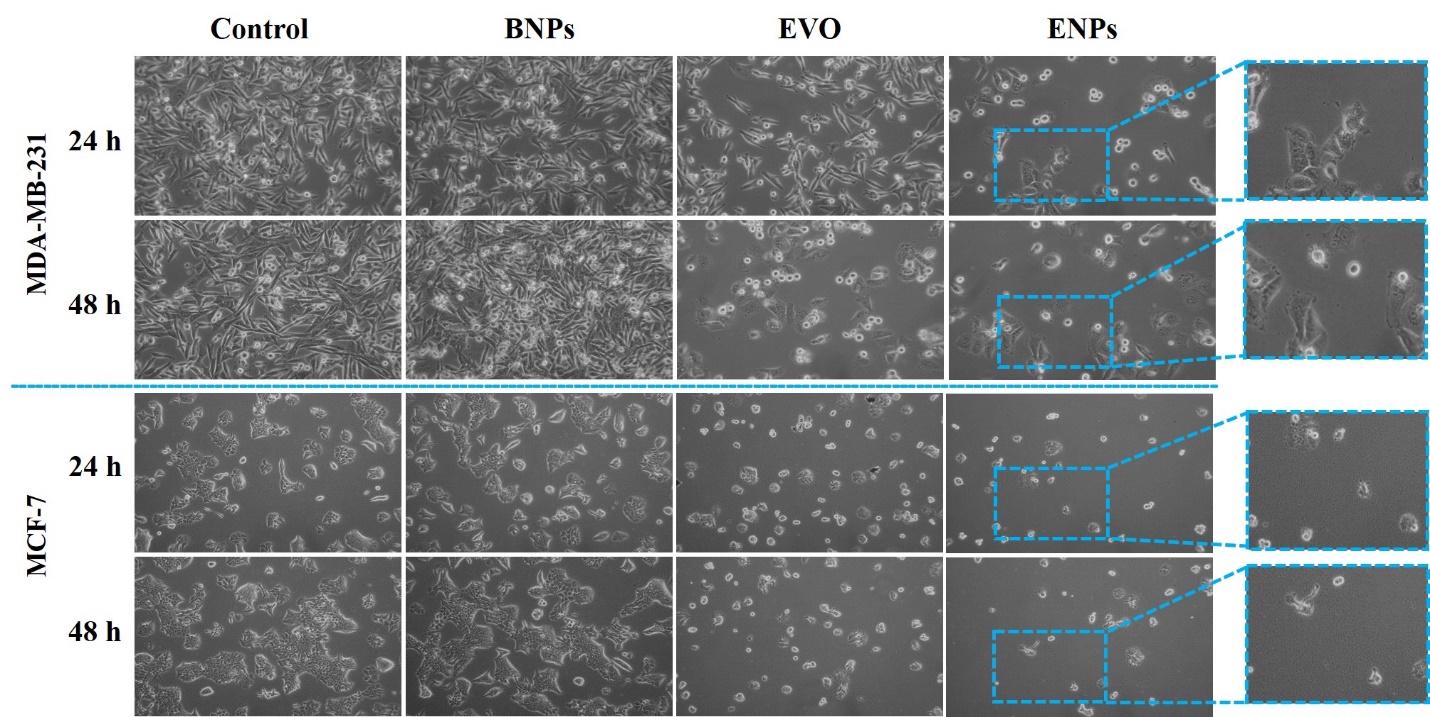


**Figure S4.** Morphological analysis of MDA-MB-231 and MCF-7 cells after treatment with EVO, BNPs and ENPs.


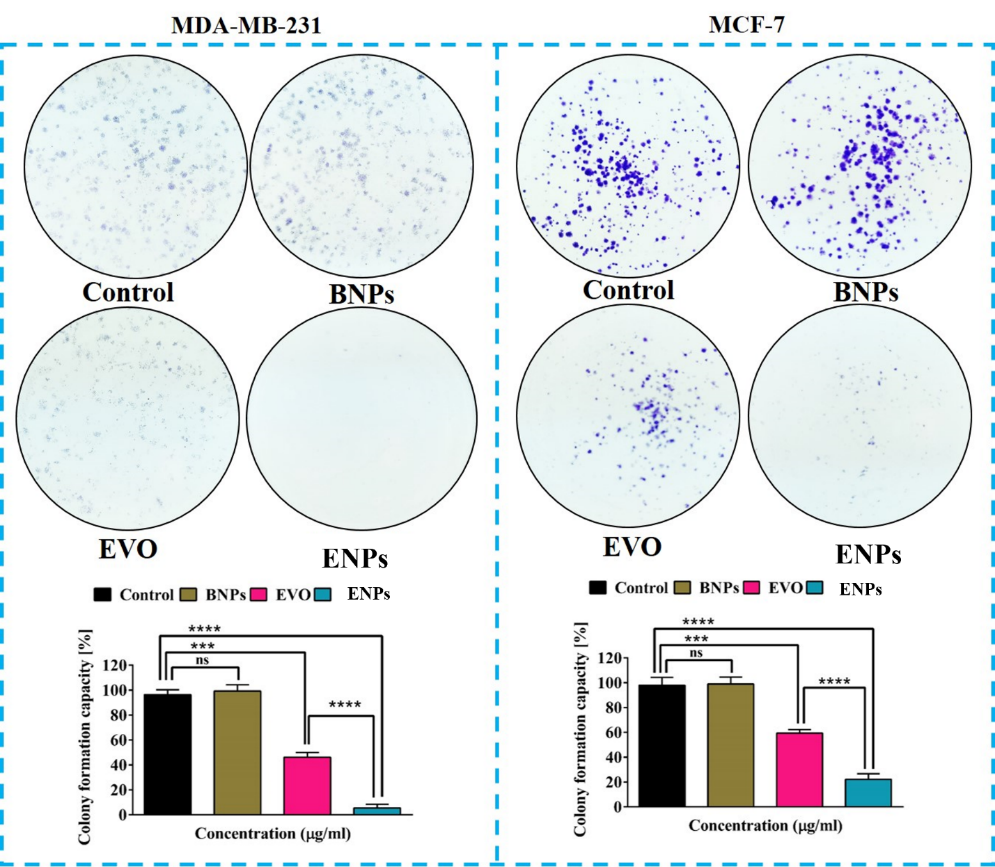


**Figure S5.** The effect of EVO, BNPs and EVO NPs on colony formation capacity in breast cancer cells.


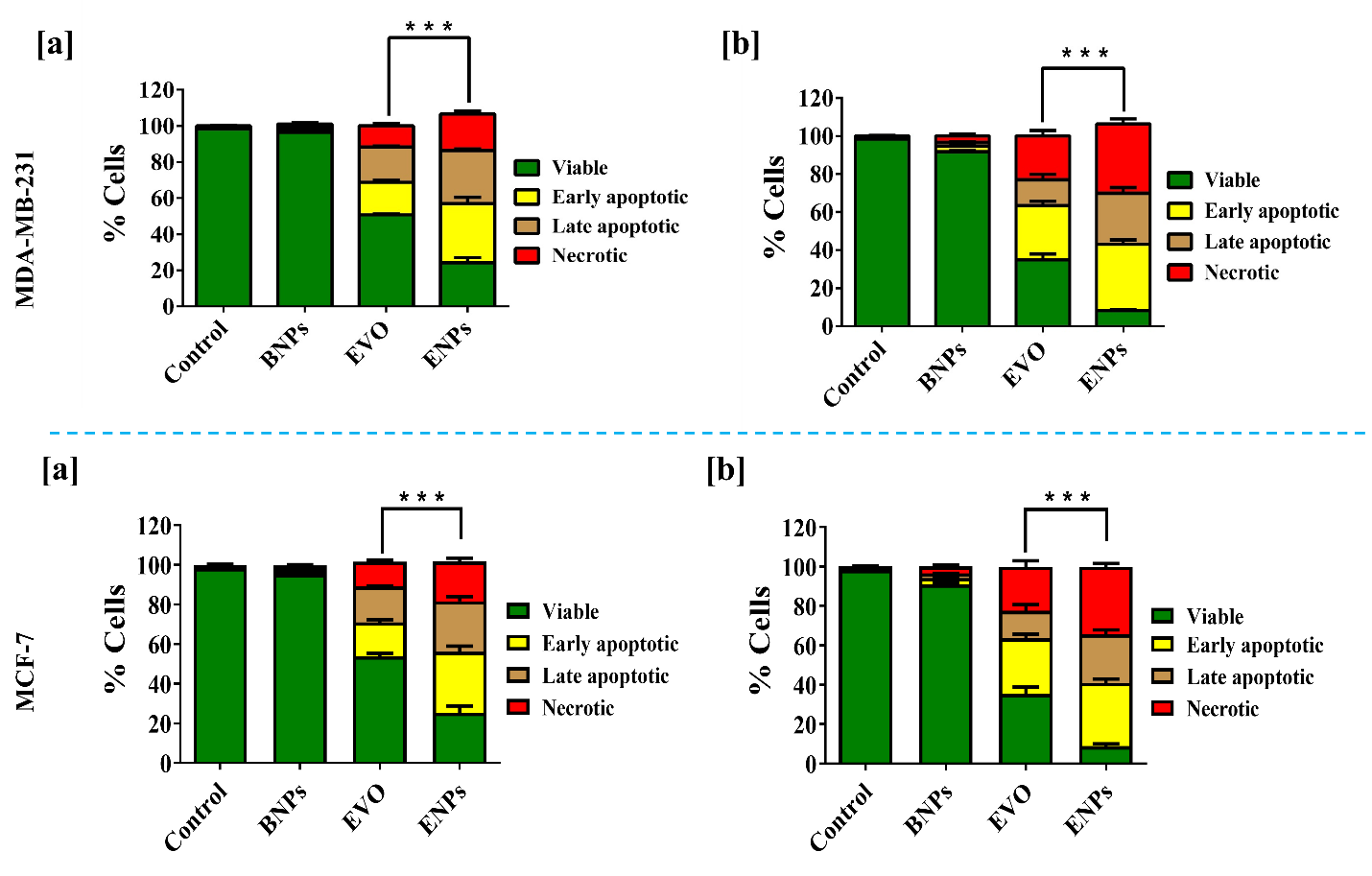


**Figure S6.** Percentage of apoptotic cells in MDA-MB-231 cells and MCF-7 cells after treatment with EVO and ENPs.


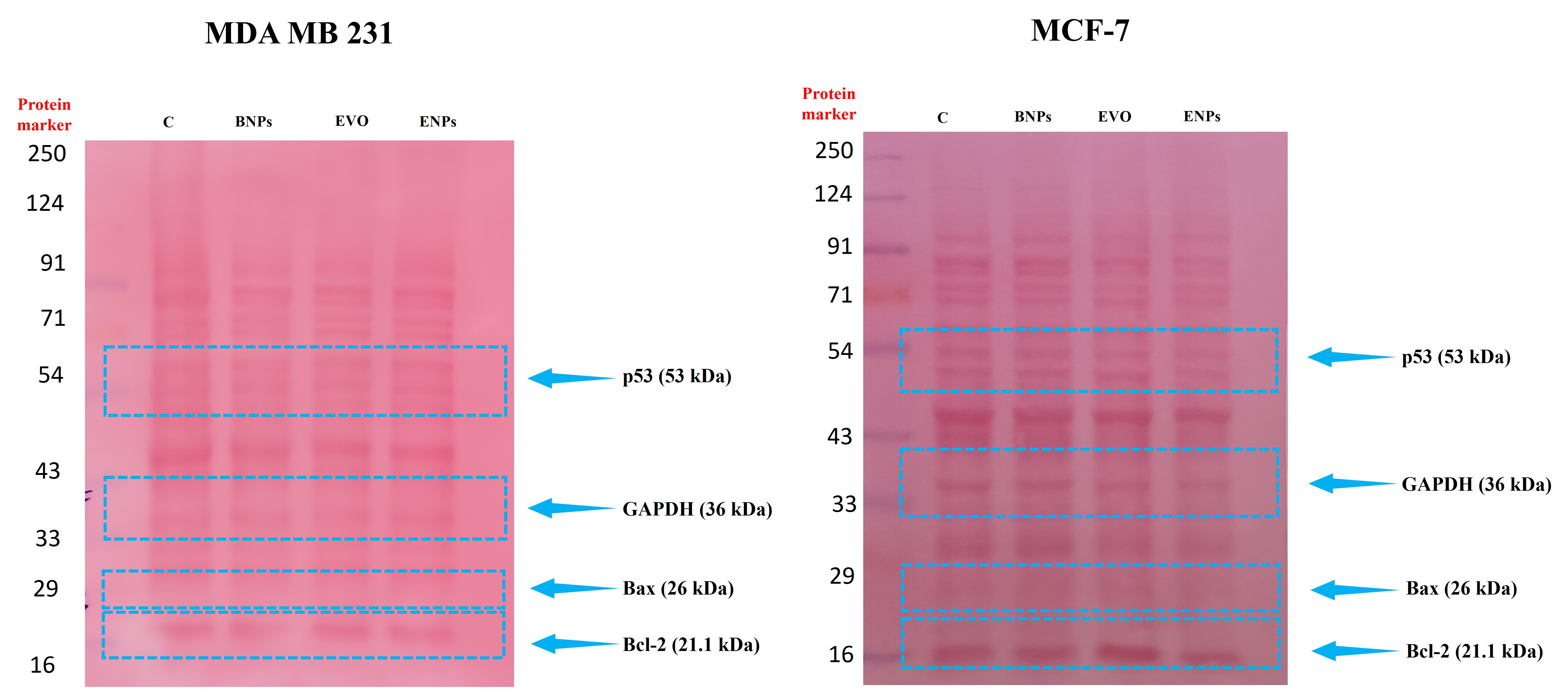


**Figure S7.** Full-length blots for MDA-MB-231 and MCF-7 cells after Ponceau Staining.


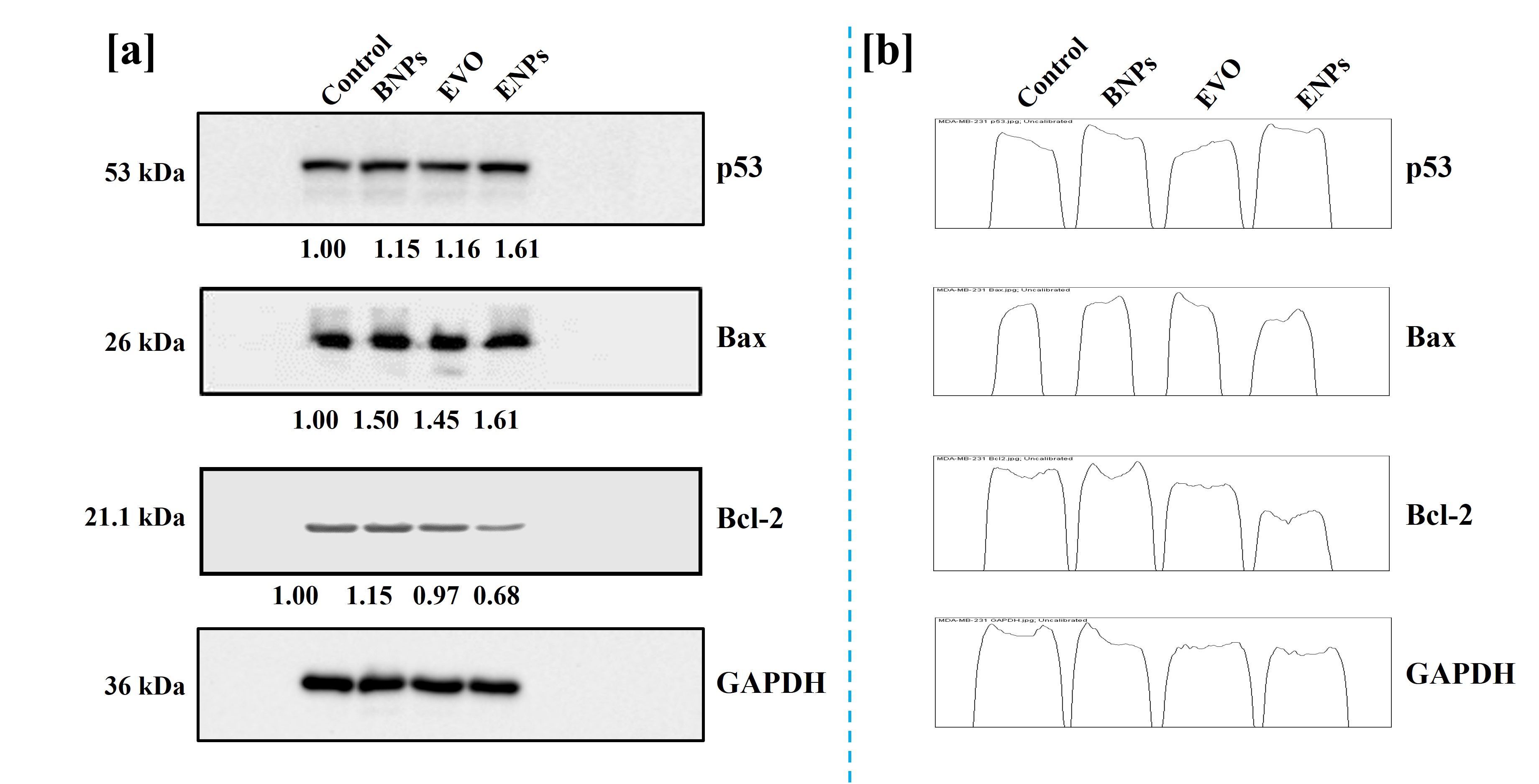


**Figure S8.** Western blot analysis. Original blots (a) and quantification graphs (b) for MDA-MB-231 cells measured by densitometry using Image J software.


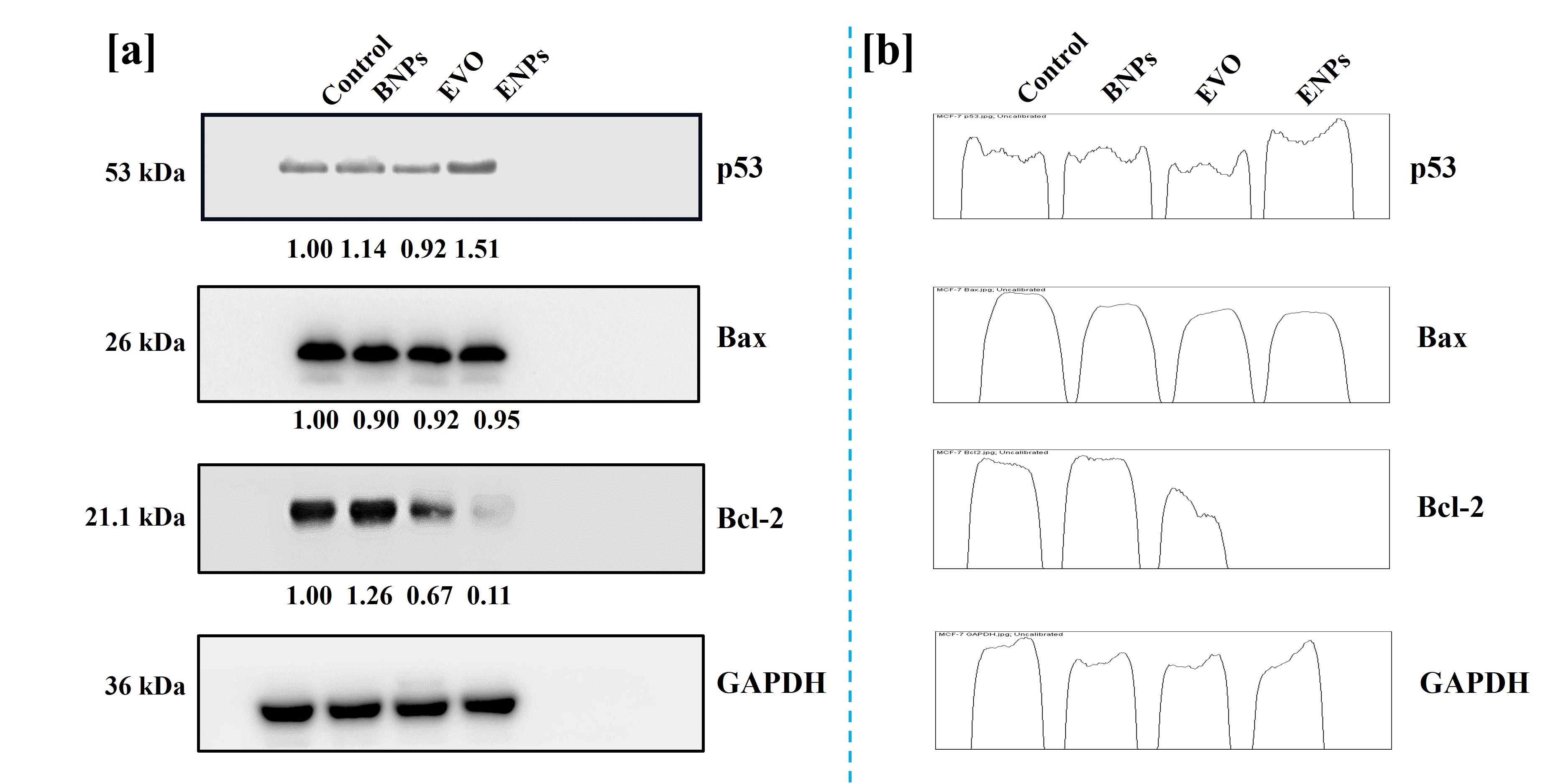


**Figure S9.** Western blot analysis. Original blots (a) and quantification graphs (b) for MCF-7 cells measured by densitometry using Image J software.


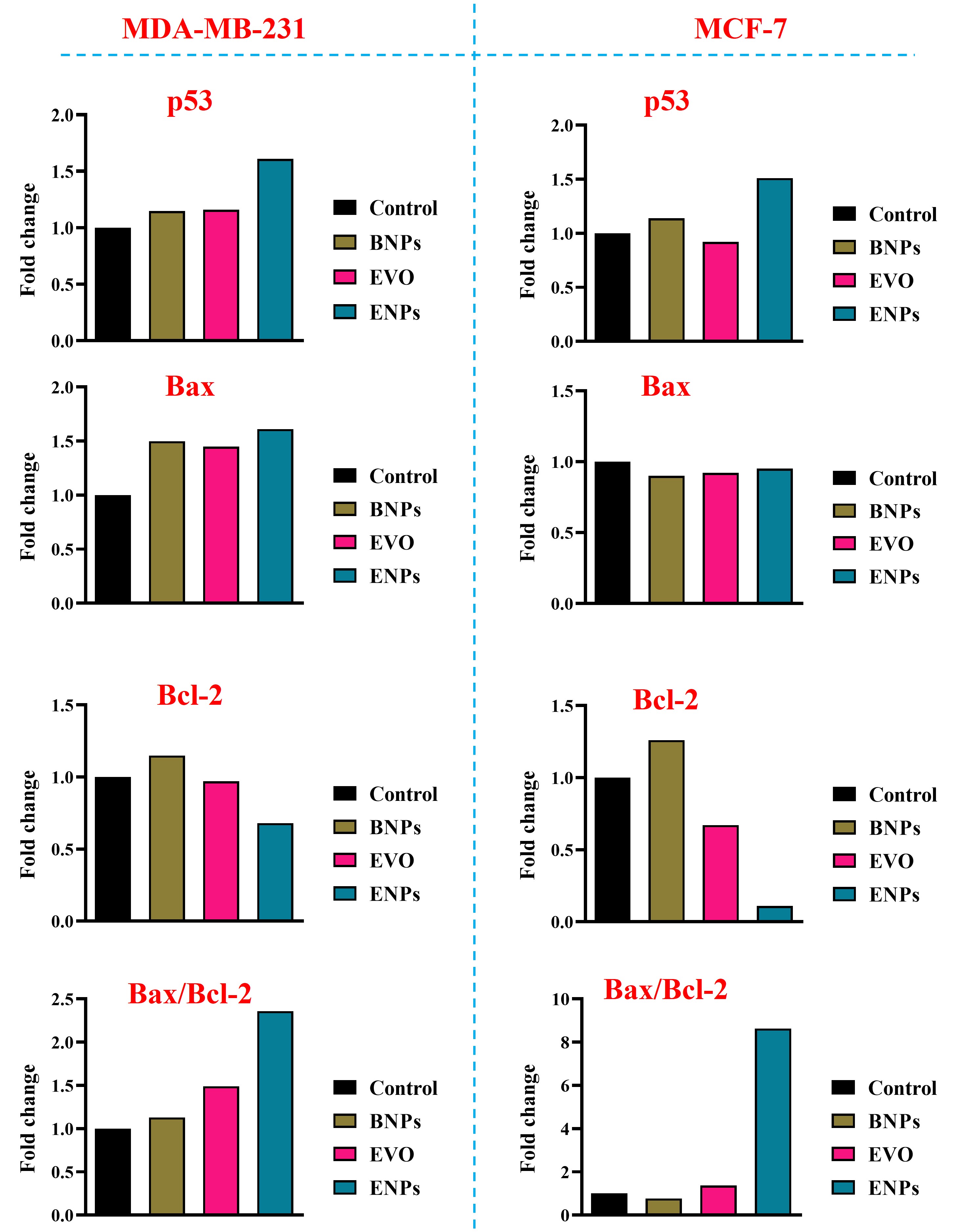


**Figure S10.** Western blot analysis. Quantification graphs of fold changes in representative blots of target proteins (p53, Bax, Bcl-2 and Bax/Bcl-2).

**Table S1.** Primer sequences. Sequences of the primers were used to quantify the gene expression levels involved in apoptosis mechanism by qRT-PCR. GAPDH was used as a reference gene.

| **Sr. No.** | **Primer** | **Sequence** |
| --- | --- | --- |
| 1 | GAPDH Forward | GACAGTCAGCCGCATCTTCT |
| 2 | GAPDH Reverse | GCGCCCAATACGACCAAATC |
| 3 | Caspase 7 Forward | GTGGGAACGATGGCAGATGA |
| 4 | Caspase 7 Reverse | GGACGGTACAAACGAGGACC |
| 5 | Caspase 9 Forward | TCAGGCCCCATATGATCGAGG |
| 6 | Caspase 9 Reverse | TCCTGGCCTGTGTCCTCTAA |
| 7 | Bax Forward | GCCCTTTTGCTTCAGGGTTT |
| 8 | Bax Reverse | TGAGACACTCGCTCAGCTTC |
| 9 | Bcl2 Forward | ATGTGTGTGGAGAGCGTCAA |
| 10 | Bcl2 Reverse | GGGCCGTACAGTTCCACAAA |
| 11 | p53 Forward | TGAAGCTCCCAGAATGCCAG |
| 12 | p53 Reverse | GCTGCCCTGGTAGGTTTTCT |
